# Predicting the earliest deviation in weight gain in the course towards manifest overweight in offspring exposed to obesity in pregnancy: a longitudinal cohort study

**DOI:** 10.1186/s12916-022-02318-z

**Published:** 2022-04-14

**Authors:** Delphina Gomes, Lien Le, Sarah Perschbacher, Nikolaus A. Haas, Heinrich Netz, Uwe Hasbargen, Maria Delius, Kristin Lange, Uta Nennstiel, Adelbert A. Roscher, Ulrich Mansmann, Regina Ensenauer

**Affiliations:** 1 Institute for Medical Information Processing, Biometry, and Epidemiology (IBE), Faculty of Medicine, Ludwig-Maximilians-Universität München, Munich, Germany; 2 Division of Pediatric Cardiology and Intensive Care, University Hospital, Ludwig-Maximilians-Universität München, Munich, Germany; 3 Department of Obstetrics and Gynecology, University Hospital, Ludwig-Maximilians-Universität München, Munich, Germany; 4 Department of General Pediatrics, Neonatology, and Pediatric Cardiology, University Children’s Hospital, Faculty of Medicine, Heinrich Heine University Düsseldorf, Düsseldorf, Germany; 5 Bavarian Health and Food Safety Authority, Oberschleißheim, Germany; 6 Department of Pediatrics, University Hospital, Ludwig-Maximilians-Universität München, Munich, Germany; 7 Institute of Child Nutrition, Max Rubner-Institut, Federal Research Institute of Nutrition and Food, Karlsruhe, Germany

**Keywords:** Maternal pre-conception obesity, Early weight gain, BMI growth, Infancy, Sequential prediction, Repeated risk assessment, Subclinical stage, Early prevention

## Abstract

**Background:**

Obesity in pregnancy and related early-life factors place the offspring at the highest risk of being overweight. Despite convincing evidence on these associations, there is an unmet public health need to identify “high-risk” offspring by predicting very early deviations in weight gain patterns as a subclinical stage towards overweight. However, data and methods for individual risk prediction are lacking. We aimed to identify those infants exposed to obesity in pregnancy at ages 3 months, 1 year, and 2 years who likely will follow a higher-than-normal body mass index (BMI) growth trajectory towards manifest overweight by developing an early-risk quantification system.

**Methods:**

This study uses data from the prospective mother-child cohort study Programming of Enhanced Adiposity Risk in CHildhood–Early Screening (PEACHES) comprising 1671 mothers with pre-conception obesity and without (controls) and their offspring. Exposures were pre- and postnatal risks documented in patient-held maternal and child health records. The main outcome was a “higher-than-normal BMI growth pattern” preceding overweight, defined as BMI *z*-score >1 SD (i.e., World Health Organization [WHO] cut-off “at risk of overweight”) at least twice during consecutive offspring growth periods between age 6 months and 5 years. The independent cohort PErinatal Prevention of Obesity (PEPO) comprising 11,730 mother-child pairs recruited close to school entry (around age 6 years) was available for data validation. Cluster analysis and sequential prediction modelling were performed.

**Results:**

Data of 1557 PEACHES mother-child pairs and the validation cohort were analyzed comprising more than 50,000 offspring BMI measurements. More than 1-in-5 offspring exposed to obesity in pregnancy belonged to an upper BMI *z*-score cluster as a distinct pattern of BMI development (above the cut-off of 1 SD) from the first months of life onwards resulting in preschool overweight/obesity (age 5 years: odds ratio [OR] 16.13; 95% confidence interval [CI] 9.98–26.05). Contributing early-life factors including excessive weight gain (OR 2.08; 95% CI 1.25–3.45) and smoking (OR 1.94; 95% CI 1.27–2.95) in pregnancy were instrumental in predicting a “higher-than-normal BMI growth pattern” at age 3 months and re-evaluating the risk at ages 1 year and 2 years (area under the receiver operating characteristic [AUROC] 0.69–0.79, sensitivity 70.7–76.0%, specificity 64.7–78.1%). External validation of prediction models demonstrated adequate predictive performances.

**Conclusions:**

We devised a novel sequential strategy of individual prediction and re-evaluation of a higher-than-normal weight gain in “high-risk” infants well before developing overweight to guide decision-making. The strategy holds promise to elaborate interventions in an early preventive manner for integration in systems of well-child care.

**Supplementary Information:**

The online version contains supplementary material available at 10.1186/s12916-022-02318-z.

## Background

Global rates of childhood obesity have increased dramatically [1]. Children with overweight or obesity are at high risk of maintaining overweight or obesity in adulthood and developing morbidities including type 2 diabetes (T2D), hypertension, and cardiovascular disease [2]. Recent evidence supports that the greatest acceleration in the child’s body mass index (BMI) growth related to sustained obesity occurs between the age of 2 and 6 years [3], suggesting that this period is critical for establishing long-term growth patterns. Before this period, in the “developmentally plastic” first 2 years of life [4], rapid postnatal weight gain has been shown to be associated with later overweight and obesity [5].

As one of the most important risk factors [6, 7], pre-conceptional maternal overweight and obesity, which affect up to 70% of pregnant women worldwide [8] and about 40% in Germany (obesity 16.4%) [9], contribute to an average 2- to 6-fold increased risk of overweight or obesity in the offspring. This effect depends on the severity of maternal obesity and the age of the child (ranging from odds ratio [OR] 2.35, 95% confidence interval [CI] 2.14–2.59 at age 2–5 years to OR 5.98, 95% CI 4.50–7.94 at age 10–18 years) [10]. During pregnancy, women with obesity are 2.5 times more likely to experience excessive gestational weight gain (GWG) [11, 12] and have a 3- to 5.5-fold higher chance of developing gestational diabetes (GDM) [13] than women with normal weight. After delivery, more than one third of mothers with overweight/obesity do not initiate breastfeeding [14], all representing specific single risk factors for childhood overweight. Circumstantial observations showed that maternal obesity and the presence of additional prenatal and/or postnatal factors, such as excessive GWG, no or short duration of breastfeeding, and unfavorable childhood eating habits, confer a substantially higher risk of overweight in offspring than maternal obesity alone [15–17]. This suggests that consideration of multiple and cumulative modifiable risk factors emerging across the very early-life span [18, 19] may help to design overweight prevention strategies for offspring of mothers with obesity.

Despite overwhelming evidence for associations of such risk factors with childhood overweight and obesity [20, 21], there is an unmet public health need to identify vulnerable infants who are at highest risk of gaining more weight than expected prior to the manifestation of overweight. Previous studies have focused on the prediction of manifest overweight/obesity in preschool and school-age children, mainly for use at a given age [22]. However, sequential prediction of the earliest deviations in weight gain patterns that precede the manifestation of overweight is not yet achieved on an individual level because of the lack of underpinning data on longitudinal BMI development and contributing predictors to develop such an approach. This would require a dynamic prediction-guided prevention strategy with serial risk assessments for “high-risk” offspring, such as those exposed to obesity in pregnancy.

In this study, we first evaluated longitudinal BMI growth patterns in offspring of mothers with obesity versus those of mothers without obesity. Secondly, a “higher-than-normal BMI growth pattern” was utilized as the endpoint, in order to define a still presymptomatic at-risk status for taking a course towards “manifest overweight.” Furthermore, we used well-documented risk associations to analyze potential contributions to the risk of developing this endpoint. The identified contributors were then condensed into a novel risk quantification system to identify those offspring from pregnancies with obesity who are at increased risk of higher-than-normal BMI growth. Finally, we embedded this prediction system into a public health approach utilizing the setting of well-child visits for early preventive interventions. We used a unique and comprehensive set of longitudinal data from the high-risk cohort Programming of Enhanced Adiposity Risk in CHildhood–Early Screening (PEACHES) of mothers with obesity and their offspring and externally validated our findings in the population-based mother-child cohort PErinatal Prevention of Obesity (PEPO).

## Methods

### Study design and populations

PEACHES is an ongoing prospective mother-child cohort study of 1671 pregnant women, mainly with obesity (*n* = 949, 56.8%), designed to investigate the long-term consequences of maternal pre-conception obesity on the development of overweight and related metabolic diseases in mothers and their offspring [23, 24]. Pregnant women were prospectively recruited during their first visit to maternity clinics (4–6 weeks before due date) in 23 hospitals mainly in the Munich area, Bavaria (southern Germany), and also in the University Hospital of Düsseldorf (western Germany) and parts of northern Germany between 2010 and 2015 [24]. Inclusion criteria in the PEACHES cohort were maternal age ≥18 years, singleton pregnancy, gestational age at birth ≥37 weeks, pre-conception BMI ≥30 kg/m^2^, and absence of preexisting type 1 diabetes (T1D) or T2D [25]. The cohort also includes mothers with normal weight, both with and without GDM, recruited as control groups [24], and a smaller proportion of overweight (and a minor number of underweight) mothers. In case the pregnancy record booklet was not ready to hand at recruitment, the mothers were re-categorized into BMI groups based on measured and recorded weight values as soon as the pregnancy record booklet became available, leading to reclassification of some women into overweight or underweight BMI categories [26], respectively. The study protocol of the PEACHES cohort was published elsewhere [24].

Data from the independent German mother-child cohort PEPO [27, 28] were used for validation. In the PEPO cohort, 11,730 children and their mothers were recruited from October 2009 to June 2011 prior to the mandatory school entry health examinations in 6 widely distributed geographical regions in Bavaria, southern Germany, both urban and rural. Inclusion criterion in the PEPO cohort was age of the child close to school entry (around 6 years). Parents and their children were invited by mail to participate via leaflets.

The local ethics committee of the Ludwig-Maximilians-Universität München, Germany, approved the cohort studies. Written informed consent was provided by all participants. The results from this study were analyzed and reported in accordance with the STrengthening the Reporting of OBservational studies in Epidemiology (STROBE) [29] and Transparent Reporting of a multivariable prediction model for Individual Prognosis Or Diagnosis (TRIPOD) [30] guidelines (Additional file 1: S1 STROBE Checklist, S2 TRIPOD Statement). Data for the analyses were retrieved from the PEACHES and PEPO databases in April 2020.

### Procedures

#### Inclusion criteria for analysis

Mothers included in the analysis were mothers with or without pre-conception obesity, were not diagnosed with T1D/T2D, and had a full-term (≥37 weeks 0 days of gestation) singleton live birth. For all analyses in each of the cohort datasets, we combined mothers with normal weight and overweight into the category “mothers without obesity” (<30 kg/m^2^), which served as a control group, as reported by others [31, 32]. Underweight women (PEACHES, *n* = 17; PEPO, *n* = 392) were excluded from the analyses.

#### Potential predictors of higher-than-normal BMI growth

In the PEACHES cohort study, data were obtained mainly from patient-held maternal and child health records (i.e., pregnancy record booklet and well-child booklet) for variables including maternal BMI at conception, total GWG, blood glucose concentrations for diagnosing GDM, parity, offspring sex and birth weight, and child anthropometric data. Data on maternal smoking during pregnancy, parental socioeconomic status (SES), and breastfeeding were gathered through questionnaires using questions from the “German Health Interview and Examination Survey for Children and Adolescents” (KiGGS) cohort study [33]. Information relating to prenatal factors was collected retrospectively shortly after delivery, mainly from documentation in the health records or via questionnaire and/or a standardized physician-administered telephone interview (e.g., smoking during pregnancy) [24]. In the PEPO cohort study, at the time of the health exam prior to primary school entry at around age 6 years, families were requested to fill out a detailed questionnaire, also containing questions from the KiGGS study [33]. In addition, trained study nurses copied all weight-related maternal and offspring data from the pregnancy record and well-child booklets, respectively.

Potential prenatal and postnatal risk predictors of higher-than-normal BMI growth were selected according to their known literature-based associations with offspring growth [34] and/or obesity [21, 35] and the availability of the data in both cohorts: maternal pre-conception BMI group, total GWG, GDM, parity, smoking during pregnancy, sex, birth weight category for gestational age and sex, SES, breastfeeding status at 1, 3, and 6 months, and offspring BMI status at the time of prediction.

Data on maternal pre-conception BMI was obtained at the time of recruitment from the pregnancy record booklet in both the PEACHES and the PEPO cohort studies. The pregnancy record booklet contains detailed information on ultrasound checkups, laboratory assessments, and weight measurements at multiple times collected by the obstetrician during antenatal care visits [24]. We used the BMI measured at the first antenatal visit as a surrogate for “pre-conception BMI” based on studies showing only a minimal difference between pre-conception weight self-reported and weight measured at the earliest antenatal visit during the first trimester [27, 36] and through own analyses (Additional file 1: Text S2, paragraph 1.1) [37]. In the PEACHES cohort, BMI was based on maternal weight and height measured (in light clothing and without shoes) by trained medical personnel at the first antenatal visit in the physicians’ offices, if the visit was before 12 weeks 6 days of gestation (PEACHES 92.4%, mean 9 weeks [SD 2 weeks] of gestation; PEPO 88.5%, mean 8 weeks [SD 2 weeks] of gestation). If the first antenatal visit was later than the 13th week of gestation (PEACHES 7.6%, PEPO 11.5%), pre-conception weight and height data as reported by the woman and documented at the first antenatal visit was abstracted from the pregnancy record booklet to calculate the pre-conception BMI.

Maternal pre-conception BMI groups were defined according to World Health Organization (WHO) categories [26] in both the PEACHES and PEPO cohort studies: normal weight (BMI 18.5 to 24.9 kg/m^2^), overweight (BMI 25.0 to 29.9 kg/m^2^), or obese (BMI ≥30.0 kg/m^2^). Mothers with obesity were further classified according to the severity of obesity, which included class 1 obesity (BMI 30.0 to 34.9 kg/m^2^), class 2 obesity (BMI 35.0 to 39.9 kg/m^2^), and class 3 obesity (BMI ≥40.0 kg/m^2^).

Total GWG was calculated using serial weight measurement data, which were documented in the pregnancy record booklet by the consulted physician throughout pregnancy [27]. Total GWG was defined as the difference between the last measured weight before delivery and pre-conception weight as defined above and was categorized as inadequate, adequate, or excessive according to the 2009 BMI-specific recommendations of the Institute of Medicine (now known as the National Academy of Medicine)/National Research Council [38].

GDM was defined as “diabetes diagnosed in the second or third trimester of pregnancy that was not clearly overt diabetes prior to gestation” [39]. All women of the PEACHES cohort who met the inclusion criteria for analysis had GDM testing by undergoing a 50-g glucose challenge test (GCT) or a 75-g oral glucose tolerance test (OGTT) during the second or third trimester of pregnancy (median 25 weeks 5 days, interquartile range [IQR] 3 weeks 1 day) [39, 40]. Diagnosis of GDM in the PEACHES cohort was based on blood glucose concentrations obtained either from the pregnancy record booklet or from laboratory test reports provided by the obstetrician. The GDM test was defined as positive when one or more of the three glucose concentrations of a 75-g OGTT met or exceeded the reference values according to the International Association of Diabetes and Pregnancy Study Groups (IADPSG) criteria (1-step procedure): fasting glucose ≥5.1 mmol/l (92 mg/dl), 1-h post-load glucose ≥10 mmol/l (180 mg/dl), or 2-h post-load glucose ≥8.5 mmol/l (153 mg/dl) [41]. In the 2-step procedure, a positive 50-g GCT (defined as 1-h post-load glucose concentration ≥7.8 mmol/l [140 mg/dl] [39]) was followed by a 75-g OGTT according to the IADPSG diagnostic criteria [41]. In the PEPO cohort, women reported the presence of GDM at the time of the school entry health examinations [42] by answering the question: “Was diabetes newly diagnosed in pregnancy prompting dietary or insulin treatment?”. At the time of the mothers’ pregnancies, GDM testing was performed between 24 weeks 0 day and 28 weeks 0 day of gestation according to the recommendations of the German Diabetes Association at the time of the study [43], which were comparable to those of the American Diabetes Association at that time [44]. All women with a diagnosis of GDM had received recommendations on treatment with insulin and/or diet, had been advised on weight gain goals, and had been monitored until the end of pregnancy by their treating physicians.

Data on maternal smoking were obtained retrospectively through two independent data sources in the PEACHES cohort (questionnaire sent to each participant and telephone interview, both carried out shortly after delivery) and by questionnaire alone in the PEPO cohort. Reported maternal smoking during pregnancy and/or the postpartum phase were categorized as “any time” versus “no time” [27].

Information on parity was abstracted from the pregnancy record booklet and categorized as primiparous (one child) or multiparous (more than one child) [45].

Data on offspring sex and birth weight were abstracted from the well-child booklets [24]. Birth weight adjusted for gestational age and sex was categorized as large-for-gestational-age (LGA; >90th percentile), average-for-gestational-age (AGA; 10th to 90th percentile), or small-for-gestational-age (SGA; <10th percentile) based on the German reference population [46].

Parental SES at birth was defined using an additive index based on maternal and paternal educational background and current type of maternal and paternal employment [47]. Information on parental education and parental employment was collected using a questionnaire, either sent to each participant in the PEACHES cohort or completed at the school entry health exam in the PEPO cohort. Educational background was categorized as “low” (<10 years of formal education [score: 1]), “medium” (10 years of formal education [score: 2]), or “high” (>10 years of formal education [score: 3]). Type of employment was categorized as “not employed” (score: 1) or “at least part-time employed” (score: 2). The parental scores of educational background and employment status were added to derive the total parental score or SES, which was categorized as “low/medium” (total parental score ≤8) or “high” (total parental score > 8).

Breastfeeding data at each time point including ages 1 month, 3 months, and 6 months were obtained retrospectively through questionnaires in both the PEACHES (at child’s ages 6 weeks and 1 year) and the PEPO cohorts and dichotomized as “not full” or “full.” “Full” includes both exclusive and predominant breastfeeding [48], where “predominant” means that the infant’s main source of nourishment during that time was breastmilk and that the infant may also have received water, water-based drinks, fruit juice, drops, or syrups [49].

#### Growth outcomes until age 5 years

In both the PEACHES and the PEPO cohort studies, child anthropometric data were abstracted from records of the regular well-child visits conducted by trained pediatricians and other professionals of the preventive health program offered to all children in Germany. In addition, anthropometric measurements were taken by trained study nurses during the school entry health exam of the PEPO children, including weight, height, and waist circumference, and carried out three times under standardized conditions [27].

In the PEACHES cohort, data from up to 9 consecutive measurements of weight and length/height were available during the first 5 years of life. These 9 measurements were collected at birth, the 1-month visit (ages 4 to 5 weeks), 3-month visit (ages 3 to 4 months), 6-month visit (ages 6 to 7 months), 1-year visit (ages 10 to 12 months), 2-year visit (ages 21 to 24 months), 3-year visit (ages 34 to 36 months), 4-year visit (ages 46 to 48 months), and 5-year visit (ages 60 to 64 months). The PEPO cohort consisted of a maximum of 4 measurements from both the well-child visits (i.e., at birth, 1-year visit, and 2-year visit) and the school entry health examination.

Consecutive age- and sex-specific BMI *z*-scores (WHO Child Growth Standards) [50, 51] were calculated to first identify (i) upper BMI growth clusters and (ii) offspring with overweight/obesity at ages 4 and 5 years, respectively. We defined offspring weight status at each time point using the WHO BMI *z*-score categorizations including >1 to ≤2 SD, >2 to ≤3 SD, >3 SD as “at risk of overweight,” “overweight,” and “obesity,” respectively, for children aged ≤60 months [51]. For children ≥61 months, we defined “overweight” and “obesity” as >1 to ≤2 SD and >2 SD, respectively. The category “BMI *z*-score >1 SD” included offspring “at risk of overweight,” with overweight, or with obesity [51]. We assumed that within a normally distributed population of offspring, 15% of offspring will be above the WHO BMI *z*-score cut-off of 1 SD [50].

Next, as the main study outcome, we used “higher-than-normal BMI growth pattern” preceding overweight, which we defined as exceeding the BMI *z*-score cut-off >1 SD at least twice in relevant offspring growth phases between 6 months and 5 years of age. Within this time window, we defined “early phase” and “late phase” as the period between 6 months to 2 years and 3 years to 5 years, respectively. Each growth phase contained three follow-up time points of BMI *z*-score assessments from well-child visits (early phase: 6-month, 1-year, and 2-year follow-ups; late phase: 3-year, 4-year, and 5-year follow-ups).

### Statistical analysis

The statistical analysis plan for all analyses can be found in the Text S1 (Additional file 1) [38, 46, 51]. We used the PEACHES cohort to search for structures and develop prediction models and performed sample size calculations to determine the appropriate size of the validation cohort. Internal and external validation was performed. Missing data were handled as missing completely at random since missing data relate to the timing of recruitment into the PEACHES cohort (i.e., offspring were too young to have their well-child follow-up at the time of data retrieval). Follow-up drop-out in offspring mainly occurred because of moving away from the study area (Additional file 1: Table S1). Given the small proportions of missing values in child follow-up data (PEACHES: 7%, PEPO: 4%, Additional file 1: Table S1), we could not identify factors related to the drop-out of participants and hence did not apply missing at random or informative missing principles.

To identify distinct BMI growth patterns from birth to 5 years of age, we performed a *k*-means cluster analysis on the longitudinal data of children of mothers with obesity (target group) and those of mothers without obesity (control group), respectively, as a non-parametric explorative analysis. We explored simultaneous effects of prenatal and postnatal factors on the endpoint “higher-than-normal BMI growth pattern” in offspring from birth to 5 years of age and during the early and late phases separately by logistic regression (including the early-phase BMI growth pattern as a potential factor influencing the late-phase pattern).

The predictive potential of prenatal and postnatal factors on the offspring’s “higher-than-normal BMI growth pattern” was examined using penalized logistic regression (least absolute shrinkage and selection operator [LASSO]) to develop consecutive prediction models and risk scoring during well-child visits at age 3 months, 1 year, and 2 years. Models were optimized according to their discriminative power (area under the receiver operating characteristic [AUROC]) by internal cross-validation. Risk scores were based on linear predictors from logistic regression. All prediction models were externally validated in the PEPO cohort. Calibration plots were based on Steyerberg et al. [52]. Data were analyzed using R software, version 3.5.1. Additional details on all statistical methods are available in the Text S2 (Additional file 1) [37, 51, 53–65]. Information on the quantification of individual risk including the development of risk scoring and risk probability assessment is provided in the Text S3 (Additional file 1).

## Results

### Characteristics of study populations

**Fig. 1:**
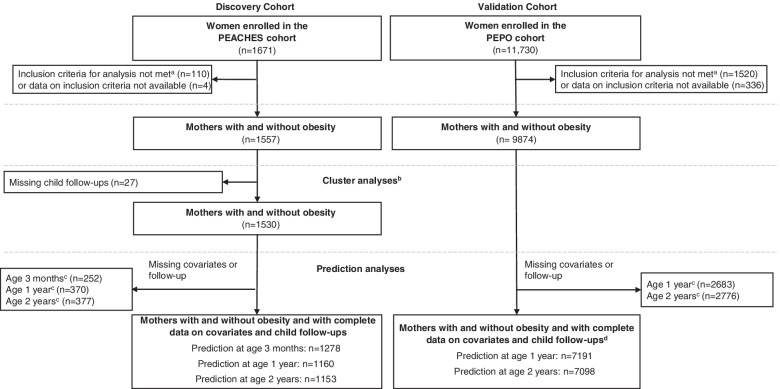
Flow chart of the study populations. ^a^Did not meet inclusion criteria for analysis, i.e., pre-conception obesity, overweight, or normal weight, full-term (≥37 weeks 0 days of gestation) singleton live birth, or absence of T1D/T2D. ^b^Identification of BMI growth clusters was not performed in the PEPO cohort due to limited offspring follow-up time points. BMI growth clusters identified in the PEACHES cohort were validated in the PEPO cohort. ^c^Missing information on at least one of the potential prenatal and postnatal predictors including maternal pre-conception BMI group, total GWG, GDM, parity, smoking during pregnancy, sex, birth weight category for gestational age and sex, SES, breastfeeding status at 1, 3, and 6 months, and/or on offspring BMI status at the respective prediction time point. ^d^External validation of prediction models at age 3 months was not performed due to unavailability of offspring BMI data at age 3 months in the PEPO cohort. BMI, body mass index; GDM, gestational diabetes; GWG, gestational weight gain; PEACHES, Programming of Enhanced Adiposity Risk in CHildhood–Early Screening; PEPO, PErinatal Prevention of Obesity; SES, socioeconomic status; T1D, type 1 diabetes; T2D, type 2 diabetes

A total of 1557 women (*n* = 887 [57.0%] with obesity and *n* = 670 [43.0%] without obesity) of the PEACHES cohort and 9874 women (*n* = 917 [9.3%] with obesity and *n* = 8957 [90.7%] without obesity) of the PEPO cohort were included in our analyses (Fig. 1, Table 1). The proportion of women excluded from the analyses due to missing data is <1% in the PEACHES cohort and 3.3% in the PEPO cohort (Fig. 1).

**Table 1:** Characteristics of the study populations

Maternal/child characteristics	Discovery cohort: PEACHES (***n*** = 1557)		Validation cohort: PEPO (***n*** = 9874)	
	Mothers with obesity (***n*** = 887)	Mothers without obesity (***n*** = 670)	Mothers with obesity (***n*** = 917)	Mothers without obesity (***n*** = 8957)
**Maternal characteristics**				
Age at conception, years	31.3 (5.3)	32.6 (5.3)	29.2 (4.9)	29.0 (5.3)
Pre-conception BMI, mean (SD), kg/m^2^	37.0 (5.2)	24.0 (3.1)	34.1 (3.8)	23.1 (2.7)
Pre-conception BMI, median (IQR), kg/m^2^	36.1 (7.3)	23.6 (4.8)	33.0 (3.8)	22.6 (4.8)
Pre-conception BMI group				
Normal weight	NA	442 (66.0)	NA	6808 (76.0)
Overweight	NA	228 (34.0)	NA	2149 (24.0)
Obese class I	384 (43.3)	NA	633 (69.0)	NA
Obese class II	278 (31.3)	NA	200 (21.8)	NA
Obese class III	225 (25.4)	NA	84 (9.2)	NA
GDM	321 (40.2)	359 (55.9)^a^	65 (7.2)	213 (2.4)
Smoking at any time during pregnancy	250 (28.4)	110 (16.7)	146 (16.2)	977 (11.1)
Primiparous	444 (50.1)	387 (57.8)	551 (62.3)	5648 (66.3)
Socioeconomic status at birth, high	244 (31.0)	408 (69.6)	125 (14.2)	2519 (29.3)
Total GWG, kg	10.9 (6.9)	13.0 (5.3)	10.3 (6.6)	13.8 (5.0)
Total GWG				
Adequate	218 (24.7)	237 (35.7)	197 (23.1)	3175 (38.4)
Excessive	512 (58.0)	237 (35.7)	486 (57.1)	3171 (38.3)
Inadequate	153 (17.3)	190 (28.6)	168 (19.7)	1925 (23.3)
**Child characteristics**				
Sex, female	420 (47.4)	351 (52.4)	450 (49.1)	4315 (48.2)
Birth weight category				
Average-for-gestational-age	690 (77.8)	520 (77.6)	684 (76.6)	7070 (81.1)
Large-for-gestational-age	101 (11.4)	45 (6.7)	130 (14.6)	677 (7.8)
Small-for-gestational-age	96 (10.8)	105 (15.7)	79 (8.8)	971 (11.1)
Full breastfeeding, at 1 month	430 (49.2)	470 (71.5)	521 (59.0)	6475 (74.4)
Full breastfeeding, at 3 months	338 (38.9)	431 (66.5)	401 (46.1)	5381 (62.2)
Full breastfeeding, at 6 months	156 (18.0)	185 (28.6)	198 (22.9)	2977 (34.8)
Child age (months) at follow-up^b^				
At 1-month follow-up	1.1 (0.2)	1.1 (0.2)	NA	NA
At 3-month follow-up	3.3 (0.5)	3.3 (0.5)	NA	NA
At 6-month follow-up	6.3 (0.7)	6.3 (0.7)	NA	NA
At 1-year follow-up	11.7 (0.9)	11.8 (0.8)	11.8 (0.8)	11.8 (0.8)
At 2-year follow-up	23.9 (1.2)	24.0 (1.2)	23.8 (1.2)	23.9 (1.1)
At 3-year follow-up	36.1 (1.1)	36.2 (1.1)	NA	NA
At 4-year follow-up	48.2 (1.4)	48.2 (1.3)	NA	NA
At 5-year follow-up	62.3 (2.2)	62.1 (1.7)	69.9 (4.8)^c^	69.7 (4.4)^c^

Values are mean (SD), median (IQR), or *n* (%). Participants with any missing information for baseline characteristics were excluded

*BMI*, body mass index; *GDM*, gestational diabetes; *GWG*, gestational weight gain; *IQR*, interquartile range; *NA*, not available; *PEACHES*, Programming of Enhanced Adiposity Risk in CHildhood–Early Screening; *PEPO*, PErinatal Prevention of Obesity

^a^The group of mothers without obesity also comprises a group of normal weight mothers with GDM, recruited as one of the control groups, as reported previously [24]

^b^Follow-up anthropometric measurements at the well-child visits

^c^Data collected at the school entry health examination, which is an obligatory check-up for children eligible to enter primary school in the coming year (before turning 6 years old) and generally takes place after the 5-year well-child visit

Baseline characteristics of mothers with and without obesity and their children are presented in Table 1. Among the mothers without obesity in the PEACHES cohort, 442 (66%) were normal weight and 228 (34%) were overweight, whereas in the PEPO cohort, 6808 (76%) were normal weight and 2149 (24%) were overweight. There was good agreement (98.7%, *n* = 1218/1234) between pre-conception weight self-reported and weight measured at the earliest antenatal visit (mean 9 weeks [SD 2 weeks] of gestation) of the PEACHES women (correlation coefficient 0.988), similar to data of the PEPO cohort [27].

Compared to control mothers without obesity, mothers with obesity in each cohort had higher percentages of LGA birth weight and shorter durations of full breastfeeding. Among women with obesity in each of the cohorts, despite having a lower mean total GWG, there was a higher proportion of excessive GWG according to the BMI-specific cut-offs [38] than among women without obesity. The proportion of GDM among women with obesity in the PEACHES cohort was 40% as in other studies [66], considering the average maternal age of > 30 years as an additional risk factor, whereas in the PEPO cohort this number was only 7.2% resulting from former less stringent criteria for diagnosing GDM [43, 44]. The mean (SD) age at child follow-up in each cohort is provided in Table 1. The median number of available follow-up data from the well-child visits was 9 (IQR 1) in the PEACHES cohort and 4 (IQR 1) in the PEPO cohort.

Data on demographic characteristics of mothers enrolled in the PEACHES and PEPO cohorts (Table 1) are similar to the German estimates, including maternal age (Germany: mean 31.6 years at birth [67]) and proportion of female offspring (Germany 48.6% [9]). The proportion of mothers belonging to a low SES at birth was 18.1% in the PEACHES cohort (Germany 20.1% [68]), whereas more women of the PEPO cohort had a low SES (32.9%). Furthermore, the proportion of children with overweight/obesity was higher in the PEACHES cohort as a total (age 3 years 7.2%) than the German (3.3%) [69] or PEPO (4.5%) estimates, based on the specific recruitment of mothers with obesity, whereas the proportions were similar when compared to only the children of mothers without obesity in the PEACHES cohort (3.2%).

### BMI growth patterns in offspring

**Fig. 2:**
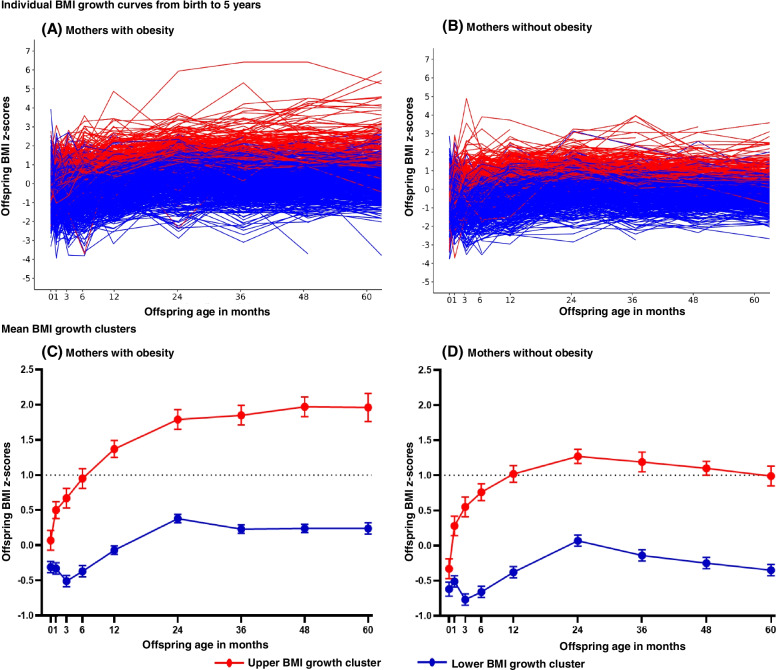
BMI growth patterns in young offspring of mothers with and without obesity. Shown are individual BMI *z*-score growth curves from birth to age 5 years in offspring of mothers with obesity (panel **A**) and without (panel **B**) enrolled in the PEACHES cohort study. Mean BMI *z*-score growth clusters along with their 95% CI are presented for offspring of mothers with obesity (panel **C**) and without (panel **D**). BMI, body mass index; PEACHES, Programming of Enhanced Adiposity Risk in CHildhood–Early Screening

The overall follow-up rate was 93% in PEACHES children and 96% in PEPO children providing 12,699 and 38,022 consecutive anthropometric measurements, respectively (Additional file 1: Table S1). Individual BMI growth patterns among PEACHES offspring of mothers with obesity (Fig. 2A) and mothers without obesity (controls) (Fig. 2B) allowed identification of two distinct BMI growth patterns from birth to 5 years of age (Fig. 2C, D, Additional file 1: Table S2). Among offspring of mothers with obesity, 21% (185/875) belonged to the upper growth cluster showing steep mean BMI *z*-score increments from birth onwards resulting in an early crossing of the WHO BMI *z*-score cut-off >1 SD at age 6 months and a growth peak at age 2 years (Fig. 2C). Across the subsequent 3 years, the mean BMI *z*-score leveled off (1.79 SD) resulting in overweight and obesity at 4 years (OR 44.56, 95% CI 20.64–96.17) and 5 years of age (OR 16.13, 95% CI 9.98–26.05).

In contrast, among 27.9% (183/655) of offspring of mothers without obesity, the upper-cluster pattern showed crossing of the mean BMI *z*-score >1 SD at 1 year of age, which appeared to decrease after peak growth at age 2 years (1.27 SD) (Fig. 2D) but also contributed to preschool overweight and obesity (age 4 years: OR 31.86, 95% CI 4.08–249.01 and age 5 years: OR 27.55, 95% CI 11.88–63.88).

Among all children belonging to upper clusters (Fig. 2C, D), those exposed to gestational obesity were at much higher risk of having multiple occasions (≥5 times) of BMI *z*-score >1 SD from age 6 months onwards (OR 5.09, 95% CI 2.99–8.68) or developing preschool overweight and obesity (age 4 years: OR 7.38, 95% CI 3.68–14.81 and age 5 years: OR 4.90, 95% CI 2.80–8.59) than offspring of mothers without obesity (Additional file 1: Figure S1) [51].

In contrast to the patterns of upper BMI growth clusters, the clusters of lower BMI growth showed similar dynamics from birth to age 2 years in the offspring of mothers with and without obesity and were below 1 SD throughout the entire period until 5 years of age (Fig. 2C, D, Additional file 1: Table S2). However, the cluster of lower BMI growth in the offspring of mothers without obesity was lower than that observed in the offspring of mothers with obesity. Among all offspring of the clusters of lower BMI growth who were older than 2 years of age, those exposed to maternal obesity in pregnancy showed a plateau in mean BMI *z*-scores, whereas offspring of mothers without obesity showed a constant reduction in mean BMI *z*-scores, i.e., before the onset of adiposity rebound. Adiposity rebound relates to a “period of dynamic changes in body composition” [70] and is equivalent to the age of the nadir of a child’s BMI curve when the BMI starts to rise again [71].

Based on the BMI growth cluster group and BMI *z*-score in the 5-year-old PEACHES offspring, we validated BMI growth clusters in the PEPO cohort for the offspring of both mothers with obesity (AUROC 0.72) and without (AUROC 0.69).

### Higher-than-normal BMI growth patterns in consecutive early-life phases

Subsequently, we found that the upper BMI growth curves of offspring with LGA (40.2%, *n* = 39/97), AGA (19.6%, *n* = 131/669), and SGA (12.8%, *n* = 12/94) birth weights from mothers with obesity (Additional file 1: Figure S2A) converged at age 3 months and continued all at a similarly high BMI growth level until age 5 years (Additional file 1: Figure S3A, Figure S3B). Comparable dynamics were seen in offspring of mothers without obesity (Additional file 1: Figure S2B, Figure S3C, Figure S3D). Based on these patterns leading to BMI convergence at 3 months and subsequent levelling off after 2 years in offspring of mothers with obesity, we determined the time points age 3 months to predict higher-than-normal BMI growth in the early phase and ages 1 year and 2 years to predict the late phase, respectively.

Maternal pre-conception obesity influenced offspring BMI growth dynamics in the transition from early to late phase, e.g., twice as many offspring of mothers with obesity developed or maintained a “higher-than-normal BMI growth pattern” when they reached the late phase (32.7%, *n* = 191/584) compared to offspring of mothers without obesity (16.8%, *n* = 72/428) (Additional file 1: Table S3) [51, 72].

### Risk factors of higher-than-normal BMI growth

**Fig. 3:**
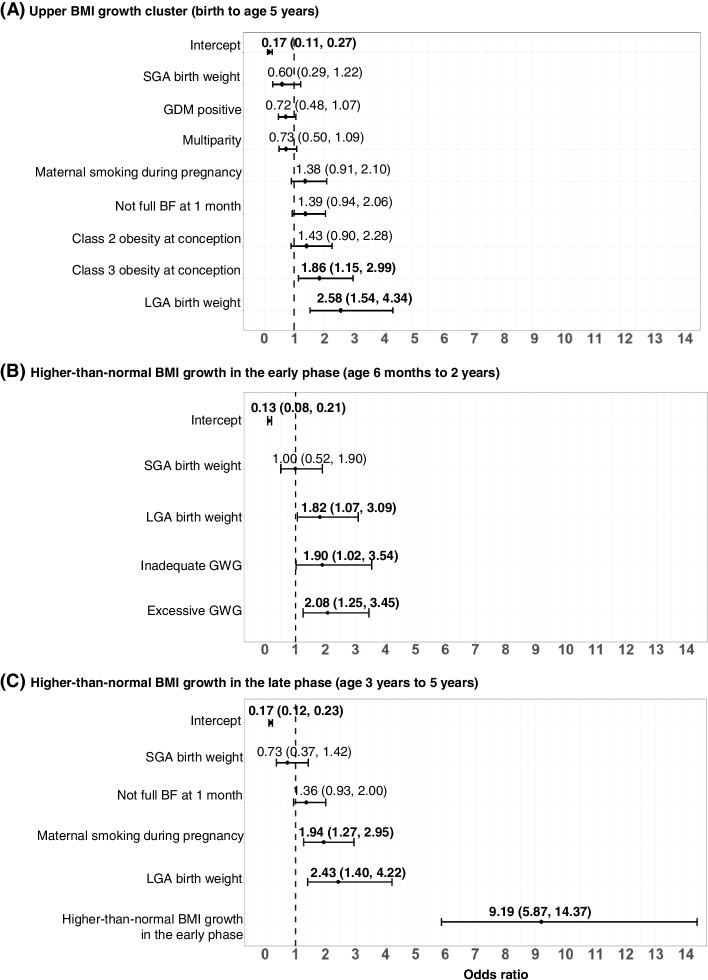
Effects of prenatal and postnatal factors on BMI growth outcomes in offspring of mothers with obesity. Shown are ORs and 95% CI of the influence of prenatal and postnatal factors on the development of an upper cluster of BMI growth (birth to age 5 years, panel **A**) and a “higher-than-normal BMI growth pattern,” defined as BMI *z*-score >1 SD [51] at least twice, during early phase (6 months to 2 years, panel **B**) and late phase (3 to 5 years, panel **C**) in offspring of mothers with obesity enrolled in the PEACHES cohort study. Values were derived from multivariable logistic regression with stepwise backward selection. Only final models based on the lowest Akaike information criterion are presented. Included variables in all initial models were maternal pre-conception BMI group, total GWG, GDM, parity, smoking during pregnancy, sex, birth weight category for gestational age and sex, SES, breastfeeding status at 1 month. Additionally, for associations shown in panel **C**, “higher-than-normal BMI growth pattern” in the early phase was also included as an explanatory variable in the initial model. BMI, body mass index; BF, breastfeeding; CI, confidence interval; GDM, gestational diabetes; GWG, gestational weight gain; LGA, large-for-gestational-age; OR, odds ratio; PEACHES, Programming of Enhanced Adiposity Risk in CHildhood–Early Screening; SES, socioeconomic status; SGA, small-for-gestational-age

Next, we assessed prenatal and postnatal factors triggering higher-than-normal BMI growth from birth until age 5 years and during early and late phases (Fig. 3, Additional file 1: Figure S4) [51]. Offspring exposed to the highest maternal pre-conception BMI in each of the groups of mothers with and without obesity were more likely to belong to the upper BMI growth cluster (Fig. 3A, Additional file 1: Figure S4A). Further, an LGA birth weight in offspring of mothers with obesity was a risk factor for a “higher-than-normal BMI growth pattern” in all growth phases studied between birth and age 5 years including both the early and late phases (Fig. 3A–C). In contrast, in offspring from pregnancies without obesity, an LGA birth weight influenced higher-than-normal BMI growth only in the early phase, not later on (Additional file 1: Figure S4B). An SGA birth weight was related to a lower BMI growth cluster in offspring of women without obesity (Additional file 1: Figure S4A). Offspring of mothers with obesity who were exposed to either excessive or inadequate GWG or smoking during pregnancy were likely to develop higher-than-normal BMI growth in the early phase or late phase, respectively (Fig. 3B, C). Lastly, regardless of maternal pre-conception obesity, higher-than-normal BMI growth in the early phase strongly triggered higher-than-normal BMI growth in the late phase (Fig. 3C, Additional file 1: Figure S4C).

### Sequential prediction of higher-than-normal BMI growth

Using these risk factors showing differential effects on BMI growth in consecutive life phases after birth, we explored and externally validated their potential to predict early-phase and late-phase “higher-than-normal BMI growth patterns” at ages 3 months, 1 year, and 2 years (Table 2, Additional file 1: Table S4) [51]. Based on these findings, we provide a workable approach for individual risk score calculations and risk probability assessment (Additional file 1: Text S3, Table S5) [51]. Risk scores above or equal to the respective cut-offs indicate risk for higher-than-normal BMI growth.

**Table 2:** Predictive performance of a sequential algorithm to identify higher-than-normal BMI growth in offspring of mothers with obesity

Predictive parameter	Prediction at age 3 months^a^	Prediction at age 1 year^b^		Prediction at age 2 years^c^	
	Higher-than-normal BMI growth in early phase (6 months–2 years)	Higher-than-normal BMI growth in late phase (3 years–5 years)		Higher-than-normal BMI growth in late phase (3 years–5 years)	
	Discovery cohort	Discovery cohort	Validation cohort	Discovery cohort	Validation cohort
*N*	711	645	670	640	666
AUROC	0.69 (0.66, 0.72)	0.73 (0.70, 0.75)	0.61	0.79 (0.76, 0.81)	0.71
Cut-off score value^d^	− 1.689	− 1.135	NA	− 1.133	NA
Prevalence, *n* (%)	140 (20.0)	194 (30.8)	221 (33.0)	192 (30.0)	223 (33.5)
Sensitivity, %	70.7 (55.5, 82.3)	73.7 (67.6, 79.0)	68.1 (62.5, 73.2)	76.0 (70.0, 81.1)	61.0 (55.3, 66.5)
Specificity, %	74.1 (61.6, 83.6)	64.7 (58.2, 70.7)	58.2 (52.5, 63.7)	78.1 (72.9, 82.5)	68.0 (63.3, 72.4)
Positive predictive value, %	40.5 (26.5, 55.6)	48.2 (41.9, 54.5)	45.3 (40.1, 50.6)	60.7 (53.5, 67.4)	49.5 (43.6, 55.3)
Negative predictive value, %	91.0 (84.7, 95.0)	84.7 (80.2, 88.3)	78.2 (73.4, 82.4)	88.0 (84.5, 90.7)	77.2 (73.3, 80.8)
Positive likelihood ratio	2.73 (1.44, 5.03)	2.09 (1.62, 2.69)	1.63 (1.32, 2.02)	3.47 (2.58, 4.64)	1.91 (1.50, 2.41)
Negative likelihood ratio	0.40 (0.21, 0.72)	0.41 (0.30, 0.56)	0.55 (0.42, 0.71)	0.31 (0.23, 0.41)	0.57 (0.46, 0.71)

We used the PEACHES cohort study as the discovery cohort and the PEPO cohort study as the external validation cohort for calculation of the individual child’s risk of a “higher-than-normal BMI growth pattern” (BMI *z*-score >1 SD [51] at least twice). Values are predictive parameters and their 95% CI

*AUROC*, area under the receiver operating characteristic; *BMI*, body mass index; *CI*, confidence interval; *GDM*, gestational diabetes; *GWG*, gestational weight gain; *NA*, not applicable; *PEACHES*, Programming of Enhanced Adiposity Risk in CHildhood–Early Screening; *PEPO*, PErinatal Prevention of Obesity; SES, socioeconomic status

^a^Potential predictors included: maternal pre-conception BMI group, total GWG, GDM, parity, smoking during pregnancy, sex, birth weight category for gestational age and sex, SES, breastfeeding status at 1 month, breastfeeding status at 3 months, and BMI *z*-score >1 SD at age 3 months. External validation of models at age 3 months could not be performed due to the lack of follow-up data at age 3 months in the validation cohort PEPO

^b^Potential predictors included: maternal pre-conception BMI group, total GWG, GDM, parity, smoking during pregnancy, sex, birth weight category for gestational age and sex, SES, breastfeeding status at 1 month, breastfeeding status at 3 months, breastfeeding status at 6 months, and BMI *z*-score >1 SD at age 1 year. External validation of models at age 1 year was performed in the validation cohort PEPO

^c^Potential predictors included: maternal pre-conception BMI group, total GWG, GDM, parity, smoking during pregnancy, sex, birth weight category for gestational age and sex, SES, breastfeeding status at 1 month, breastfeeding status at 3 months, breastfeeding status at 6 months, and BMI *z*-score >1 SD at age 2 years. External validation of models at age 2 years was performed in the validation cohort PEPO

^d^Offspring with a risk score above or equal to the respective cut-off score value are considered to be at risk of developing a “higher-than-normal BMI growth pattern.” The cut-off value of the score was optimized to avoid false-negative findings (sensitivity), which resulted in negative cut-off score values

The score based on the first risk quantification model applicable at age 3 months allowed a good prognosis of early-phase growth, and the cut-off value was optimized to avoid false-negative findings: 70.7% of offspring of mothers with obesity with scores ≥− 1.689 will develop higher-than-normal BMI growth in the early phase (sensitivity) and 74.1% with scores <− 1.689 will not (specificity) (Table 2). The positive predictive value indicates that 40.5% of all offspring of mothers with obesity identified as “at risk” at age 3 months will certainly develop a “higher-than-normal BMI growth pattern” in the early phase, and the negative predictive value indicates that 91.0% of all offspring of mothers with obesity classified as being “not at risk” will indeed not develop a “higher-than-normal BMI growth pattern.” Furthermore, the positive likelihood ratio value of 2.73 indicates an increase (15%) [57] in the likelihood of developing a “higher-than-normal BMI growth pattern” in offspring identified as “at risk” by the prediction model at age 3 months. The negative likelihood ratio value of 0.40 indicates a decrease (20%) [57] in the likelihood of developing a “higher-than-normal BMI growth pattern” in offspring identified as “not at risk” by the same prediction model at 3 months of age.

The subsequent prediction models at ages 1 year and 2 years developed for risk re-assessments for the late phase showed even higher predictive performance (Table 2). While the negative predictive values were similarly high for all models, the predictive model at age 2 years had the highest sensitivity, specificity, positive predictive value, and positive likelihood ratio and the lowest negative likelihood ratio. Similarly good parameters were observed in offspring of mothers without obesity (Additional file 1: Table S4).

In the independent validation cohort PEPO, prediction models using available data at ages 1 year and 2 years showed fair predictive performances in offspring of both mothers with and without obesity (Table 2, Additional file 1: Table S4). Furthermore, prediction models at age 1 year and 2 years for offspring of mothers with obesity and at age 1 year for offspring of mothers without obesity showed good and very good calibration (i.e., agreement between observed and predicted risks), respectively, using the external cohort PEPO (Additional file 1: Figure S5) [51]. Details on use of individual risk score calculations, risk probability assessment, and clinical case scenarios are provided in Table S5 and the Text S3 (Additional file 1).

## Discussion

Longitudinal data from two large mother-child cohorts led to the identification of a “high-risk” subpopulation of offspring susceptible to early upper deviations from healthy weight gain trajectories and novel risk stratification in the very first “plastic phase” of life. Such a strategy could allow cost-effective and personalized advice and measures to slow down or prevent otherwise ongoing increases in BMI growth. Several modifiable influences associated with gestational overnutrition, such as grade of maternal obesity at conception, excessive GWG, and LGA birth weight, contributed sequentially during consecutive early phases to the offspring’s susceptibility to gain more weight than expected. Here, these already well-documented risk associations were translated and condensed into a novel serial prediction strategy for primary prevention of a “higher-than-normal BMI growth pattern” as a subclinical stage preceding overweight in clinical settings. The system of well-child visits is ideal for identifying risks by the pediatricians and providing targeted supportive measures to guide offspring BMI growth from early life onwards.

The 2- to 3-fold increased risk of overweight/obesity even in young children from pregnancies with obesity below age 5 years [10] prompted us to study the type and potential predictors of very early growth patterns towards overweight. Unlike previous studies [31, 73], we focused on identifying such growth patterns among offspring exposed to an adipogenic intrauterine milieu. The identified BMI growth pattern showing recurrent crossing of BMI *z*-scores >1 SD—the WHO cut-off for “at risk of overweight”—from an early age of 6 months is highly likely to set the stage for developing overweight at preschool age, which is critical to sustained obesity [3]. The upper BMI growth cluster in obesity-exposed offspring plateaued at high levels after age 2 years. This levelling off is in contrast to the typical BMI decline [71] preceding the adiposity rebound at around 6 years [74]. Our findings suggest that obesity in pregnancy could potentially “hit” cellular processes in the fetus, which may influence offspring outcomes such as postnatal appetite regulation and fat accretion before overweight manifestation [75].

Besides maternal pre-conception obesity [34, 76], offspring weight development can be shaped by additional influences highly associated with an obesogenic environment during prenatal and postnatal life [77, 78]. We found that among such obesity-associated factors, those relating to intrauterine overnutrition including excessive GWG contributed strongly to higher-than-normal BMI growth during the “plastic phase” of the first 2 years. Indeed, there is a need for women with obesity to be provided with more customized advice on dietary intake and physical activity to optimize gestational weight management [24, 79]. For the subsequent time period of the preschool years, gestational smoking emerged as a relevant modulator of growth in offspring exposed to obesity in pregnancy in our study. This association may take time to appear since mothers who smoke at the beginning and/or later during pregnancy are likely to resume smoking postnatally and therefore refrain from breastfeeding more frequently [80, 81], predisposing offspring to develop overweight [82]. In mothers with obesity of the PEACHES cohort study who had smoked during pregnancy, any smoking (versus no smoking) within the first weeks postpartum was related to higher odds of stopping full breastfeeding by the end of the first month (OR 1.97, 95% CI 1.10–3.53). Irrespective of smoking, mothers with obesity have been recognized to experience major difficulties with initiating and continuing breastfeeding resulting in lower breastfeeding rates in these women [83]. Supporting previous evidence [75], our data also show the relevance of an LGA birth weight for overweight development, irrespective of maternal pre-conception BMI, albeit it seemed to have adverse longer-term consequences only in children of mothers with obesity. Our data point to the differential contribution of “obesogenic influences” arising from the pre-gestational, gestational, and perinatal periods, such as grade of maternal obesity at conception, excessive weight gain and smoking during pregnancy, as well as LGA birth weight on higher-than-normal BMI growth during successive early-life phases after birth.

**Fig. 4:**
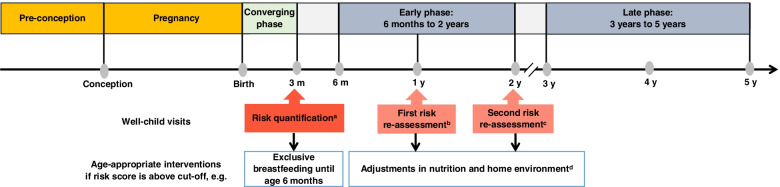
Prediction-guided prevention strategy for the risk of developing a “higher-than-normal BMI growth pattern” preceding overweight. “Higher-than-normal BMI growth pattern” defined as BMI *z*-score >1 SD [51] at least twice in relevant growth phases from 6 months to 5 years. ^a^Initial risk quantification is performed at the 3-month well-child visit for “higher-than-normal BMI growth pattern” during the early phase (6 months to 2 years). ^b^First risk re-assessment is performed at the 1-year visit for “higher-than-normal BMI growth pattern” during the late phase (3 to 5 years). ^c^Second risk re-assessment is performed at the 2-year visit for “higher-than-normal BMI growth pattern” during the late phase (3 to 5 years). ^d^Overweight-preventive measures such as healthier complementary and family food choices, reduced screen time, increased physical activity, and a sleep duration of 10 to 14 h per day. If a risk score is above or equal to the respective cut-off score value (Table 2, Additional file 1: Table S4), the child is at risk of developing a “higher-than-normal BMI growth pattern,” and age-appropriate obesity-preventive measures should be initiated by the pediatrician. BMI, body mass index; h, hours; m, months; y, year(s)

Using these modifiable factors [84], we developed a novel strategy to identify infants likely to deviate from the normal BMI growth pattern as a subclinical stage before establishing preschool overweight. Unlike previous methods that offered prediction of manifest overweight [56, 85] and/or were applicable at a certain age only [22] and were developed for offspring born to women of heterogeneous BMI [22], we propose a novel sequential strategy of prediction and re-evaluation of higher-than-normal weight gain in “high-risk” offspring of mothers with obesity at ages 3 months, 1 year, and 2 years to guide pediatric decision-making (Fig. 4). Owing to these differences in the outcome (“higher-than-normal BMI growth pattern”), population (offspring of mothers with obesity), and prediction time points (sequential prediction) between our and previous work, prediction models cannot be directly compared. Integrating such a novel dynamic element in the existing health care system of well-child visits could help to quantify and confine risk to subpopulations and individuals at high necessity to intervene. These preventive visits have a high participation rate [86], even up to 99% of children in Germany, and take place seven times during the first 2 years of life [87]. Interventions to optimize BMI development during the first 1000 days are more beneficial than during preschool ages [88], as an “adaptive phase” when offspring have a chance of returning to their “genetic growth potential” [89].

Thus, following risk stratification by individual risk score and probability calculation at the 3-month well-child visit, breastfeeding continuation can be reinforced by the pediatrician, given the protective role of breastfeeding, e.g., in overweight prevention [82]. Considering the generally low exclusive breastfeeding rates at 6 months (Europe 25% [90]; Germany 12.5% [91]) and the lack of effective intervention strategies to increase the rate and duration of breastfeeding particularly in mothers with obesity [92], prediction-guided “individualized” breastfeeding support by prescribing extra lactation counselling beyond standard care seems promising. Following risk prediction at the 1-year and 2-year visits, mothers with obesity and “high-risk” children may benefit from specific dietary counselling by nutritionists to encourage healthy complementary and family food choices, since early eating patterns determine future eating habits and the development of childhood overweight/obesity [93].

Typical for a screening setting, our prediction models show high sensitivity to avoid false-negative cases and high negative predictive values to avoid misclassification as being “not at risk” in offspring with higher-than-normal BMI growth. Furthermore, even a high false-positive rate, i.e., identifying offspring with normal BMI development as being “at risk,” can be considered acceptable since obesity-preventive interventions including exclusive breastfeeding [82] and improved nutrition (such as healthier complementary and family food choices) as well as supportive environments (such as reducing screen time, increasing physical activity, maintaining a sleep duration of 10 to 14 h per day) [94–96] (Fig. 4) are beneficial and safe for young children’s growth in general. However, targeting such interventions to a defined subpopulation of offspring at need will direct resources, i.e., costs for personal counselling, to those at the highest risk of excessive BMI growth and help minimizing health care costs.

The strength of our study is the large contemporary prospective mother-child cohort PEACHES of 1671 mothers and children providing a unique longitudinal dataset with wide-ranging pre-, peri-, and postnatal variables from mothers with obesity, and thus, it was used as the discovery cohort. Multiple anthropometric measurements improved precision to identify minor deviations in BMI growth especially in the sensitive first months of life. Based on the time structure of the data and use of robust machine learning techniques [97, 98], our proposed strategy provides multiple prediction occasions within an early window of opportunity for prevention of higher-than-normal BMI growth, utilizing routinely available data and making it easy-to-use in clinical settings [99]. Internal (cross-validation) and external (PEPO cohort) validation showed good discrimination between higher-than-normal and normal BMI growth in offspring of mothers either with or without obesity. Attrition bias is unlikely as the follow-up rates in offspring were around 95% in both the PEACHES and PEPO cohorts. The prediction models performed sufficiently well and showed good to very good calibration for early-risk stratification and identification of “high-risk” offspring.

Minor differences between the two cohorts relating to the recruitment strategy, the proportion of mothers with pre-conception obesity, and offspring follow-up time points could influence the lower predictive potential of models in the PEPO cohort. However, despite the differences, external validation showed adequate predictive performance and indicates robustness of our results. Furthermore, we aimed at developing discriminative models for offspring of mothers with and without obesity separately and did not recalibrate the models when applied to the external cohort PEPO. Nevertheless, the predictive models require recalibration when applied to other populations. For prediction models relating to offspring of mothers without obesity, results may not be comparable to other studies with a different composition in the proportions of mothers with normal weight and overweight. Still, we were able to confirm our findings in the PEPO cohort. Regarding the association analyses, we used literature-based risk associations for manifest overweight and applied them to the endpoint “higher-than-normal BMI growth pattern” to test whether there is evidence for an association based on a qualitative approach. Therefore, we did not correct model coefficients by specific shrinking techniques. However, for the development of our prediction score, this was accounted for using penalized regression strategies.

Future studies should develop a user-friendly tool for risk score calculations and evaluate prospectively whether the proposed prediction strategy is effective in guiding favorable BMI growth in early childhood. Such a tool should be easy-to-use in clinical practice, and results should be communicated in an informative manner [100, 101], e.g., a web-based Shiny application developed using the Shiny R package for building easy and interactive web apps in R [102]. Implementing such an instrument and designing a prospective validation study are plans for our future research.

## Conclusion

In conclusion, based on a unique set of validated longitudinal data on BMI outcomes in offspring exposed to obesity in pregnancy, we identified a population of offspring at highest risk of an early-starting higher-than-normal BMI growth trajectory inevitably followed by overweight. For individual risk quantification, we devised a novel sequential prediction system to allow early-risk stratification and re-evaluation for prevention of a “higher-than-normal BMI growth pattern” as a subclinical stage preceding overweight. Our proposed prediction strategy could stimulate the use of cost-effective and personalized advice and measures counteracting the risk of very early excess weight gain. Integrating such a procedure in the existing health care systems of well-child visits could help to quantify and confine risk to subpopulations and individuals at high necessity to intervene.

## Supplementary Information

**Additional file 1: S1** STROBE Checklist. **S2** TRIPOD Statement. **Text S1.** Statistical analysis plan. **Text S2.** Statistical methods. **Text S3.** Quantification of individual risk. **Figure S1.** Influence of maternal obesity on offspring BMI growth outcomes. Shown are ORs and 95% CIs of the influence of maternal pre-conception obesity on BMI growth outcomes up to age 5 years in all offspring belonging to upper BMI growth clusters from the PEACHES cohort study. Values were derived from univariate logistic regression. ^a^The term “multiple occasions” was defined as having BMI z-scores >1 SD [51] at least 5 out of 6 times at the well-child visits at age 6 months, 1 year, 2 years, 3 years, 4 years, and 5 years. BMI, body mass index; CI, confidence interval; OR, odds ratio; PEACHES, Programming of Enhanced Adiposity Risk in CHildhood–Early Screening. **Figure S2.** Proportion of offspring in upper and lower BMI growth clusters according to birth weight category. Shown are percentages in offspring of mothers with obesity (panel A) and without (panel B) enrolled in the PEACHES cohort study, according to their birth weight category for gestational age and sex. AGA, average-for-gestational-age; BMI, body mass index; LGA, large-for-gestational-age; PEACHES, Programming of Enhanced Adiposity Risk in CHildhood–Early Screening; SGA, small-for-gestational-age. **Figure S3.** Mean BMI growth clusters by birth weight category in offspring of mothers with and without obesity. Shown are mean BMI z-score growth clusters from birth to age 6 months (panel A, C) and birth to age 5 years (panel B, D) by birth weight category for gestational age and sex in offspring of mothers with and without obesity enrolled in the PEACHES cohort study. AGA, average-for-gestational-age; BMI, body mass index; LGA, large-for-gestational-age; PEACHES, Programming of Enhanced Adiposity Risk in CHildhood–Early Screening; SGA, small-for-gestational-age. **Figure S4.** Effects of prenatal and postnatal factors on BMI growth outcomes in offspring of mothers without obesity. Shown are ORs and 95% CI of the influence of prenatal and postnatal factors on the development of an upper cluster of BMI growth (birth to age 5 years, panel A) and a “higher-than-normal BMI growth pattern,” defined as BMI z-score >1 SD [51] at least twice, during early phase (6 months to 2 years, panel B) and late phase (3 years to 5 years, panel C) in offspring of mothers without obesity enrolled in the PEACHES cohort study. Values were derived from multivariable logistic regression with stepwise backward selection. Only final models based on the lowest Akaike information criterion are presented. Included variables in all initial models were maternal pre-conception BMI group, total GWG, GDM, parity, smoking during pregnancy, sex, birth weight category for gestational age and sex, SES, breastfeeding status at 1 month. Additionally, for associations shown in panel C, “higher-than-normal BMI growth pattern” in the early phase was also included as an explanatory variable in the initial model. BMI, body mass index; CI, confidence interval; GDM, gestational diabetes; GWG, gestational weight gain; LGA, large-for-gestational-age; OR, odds ratio; PEACHES, Programming of Enhanced Adiposity Risk in CHildhood–Early Screening; SES, socioeconomic status; SGA, small-for-gestational-age. **Figure S5.** Calibration plots of prediction models for identifying a “higher-than-normal BMI growth pattern” in the validation cohort. Shown are calibration curves (blue lines) and calibration slopes and intercepts for offspring of mothers with obesity (panel A, B) and without obesity (panel C, D) by the prediction models at age 1 year and 2 years. The diagonal gray lines represent the optimal prediction; the closer the model curve is to the diagonal line, the more accurate is the prediction. At the top of each graph, dots indicate presence of the outcome “higher-than-normal BMI growth pattern,” defined as BMI z-score >1 SD [51] at least twice, in the late phase (3 years to 5 years). At the bottom of each graph, dots indicate absence of the outcome “higher-than-normal BMI growth pattern” in the late phase. Calibration of models at age 3 months for “higher-than-normal BMI growth pattern” in the early phase (6 months to 2 years) could not be performed due to the lack of follow-up data at age 3 months in the validation cohort PEPO. BMI, body mass index; PEPO, PErinatal Prevention of Obesity. **Table S1.** Offspring follow-up rates in the study populations. Values are n (%). ^a^Missing data in the PEACHES cohort were due to loss to follow-up. Missing data in the PEPO cohort were due to lack of availability of data in the records of the regular well-child visits at the time of school entry health examination. ^b^A total of 13 and 297 children enrolled in the PEACHES cohort were too young for the follow-up visit at age 4 and 5 years, respectively, and therefore were not included in the “total” category. Missing data were considered missing completely at random. NA, not available; PEACHES, Programming of Enhanced Adiposity Risk in CHildhood–Early Screening; PEPO, PErinatal Prevention of Obesity. **Table S2.** Mean BMI z-scores by BMI growth cluster in offspring of mothers with and without obesity. Values are mean and 95% CI in offspring of mothers with and without obesity enrolled in the PEACHES cohort study. ^a^Of a total of 887 children included for cluster analysis, 875 children could be categorized into longitudinal BMI growth clusters based on an adequate number of data points. ^b^Of a total of 670 children included for cluster analysis, 655 children could be categorized into clusters based on an adequate number of data points. ^c^A total of 276 and 549 children enrolled in the PEACHES cohort were not included in the cluster analysis at age 4 and 5 years, respectively, because of follow-up not yet due (age 4 years: n=13, age 5 years: n=297) or missing data due to loss to follow-up (age 4 years: n=263, age 5 years: n=252). Missing data were considered missing completely at random. BMI, body mass index; CI, confidence interval; PEACHES, Programming of Enhanced Adiposity Risk in CHildhood–Early Screening. **Table S3.** Offspring BMI growth dynamics in consecutive life phases after birth following exposure to gestational obesity. Values are n (%) in offspring enrolled in the PEACHES cohort study. Only children with complete data on BMI z-scores in both the early and late phase are presented. ^a^Includes values for categories “normal range” (≥-2 to ≤1 SD) and a minor proportion of children with <-2 SD [72]. ^b^BMI z-score >1 SD defined as occurring once. Includes values for categories “at risk of overweight” (>1 to ≤2 SD), overweight (>2 to ≤3 SD), and obesity (>3 SD) [51]. ^c^“Higher-than-normal BMI growth pattern” defined as BMI z-score >1 SD [51] at least twice. BMI, body mass index; PEACHES, Programming of Enhanced Adiposity Risk in CHildhood–Early Screening. **Table S4.** Predictive performance of a sequential algorithm to identify higher-than-normal BMI growth in offspring of mothers without obesity. We used the PEACHES cohort study as the discovery cohort and the PEPO cohort study as the external validation cohort for calculation of the individual child’s risk of a “higher-than-normal BMI growth pattern” (BMI z-score >1 SD [51] at least twice). Values are predictive parameters and their 95% CI. ^a^Potential predictors included: maternal pre-conception BMI group, total GWG, GDM, parity, smoking during pregnancy, sex, birth weight category for gestational age and sex, SES, breastfeeding status at 1 month, breastfeeding status at 3 months, and BMI z-score >1 SD at age 3 months. External validation of models at age 3 months could not be performed due to the lack of follow-up data at age 3 months in the validation cohort PEPO. ^b^Potential predictors included: maternal pre-conception BMI group, total GWG, GDM, parity, smoking during pregnancy, sex, birth weight category for gestational age and sex, SES, breastfeeding status at 1 month, breastfeeding status at 3 months, breastfeeding status at 6 months, and BMI z-score >1 SD at age 1 year. External validation of models at age 1 year was performed in the validation cohort PEPO. ^c^Potential predictors included: maternal pre-conception BMI group, total GWG, GDM, parity, smoking during pregnancy, sex, birth weight category for gestational age and sex, SES, breastfeeding status at 1 month, breastfeeding status at 3 months, breastfeeding status at 6 months, and BMI z-score >1 SD at age 2 years. External validation of models at age 2 years was performed in the validation cohort PEPO. ^d^Offspring with a risk score above or equal to the respective cut-off score value are considered to be at risk of developing a “higher-than-normal BMI growth pattern.” The cut-off value of the score was optimized to avoid false-negative findings (sensitivity), which resulted in negative cut-off score values. AUROC, area under the receiver operating characteristic; BMI, body mass index; CI, confidence interval; GDM, gestational diabetes; GWG, gestational weight gain; NA, not applicable; PEACHES, Programming of Enhanced Adiposity Risk in CHildhood–Early Screening; PEPO, PErinatal Prevention of Obesity; SES, socioeconomic status. **Table S5.** Scoring system for quantification of risk of higher-than-normal BMI growth in young offspring. ^a^The equations can be used for sequential individual risk quantification of a “higher-than-normal BMI growth pattern” (BMI z-score >1 SD [51] at least twice) in offspring of mothers with or without pre-conception obesity separately. The prenatal and postnatal variables in the risk quantification equations should be replaced by pre-defined values (0 or 1) depending on whether the condition stated is fulfilled (1) or not (0). The calculated risk score should be compared to the respective cut-off score value (Table 2, Additional file 1: Table S4) . Offspring with a risk score above or equal to the respective cut-off are considered to be at risk of developing a “higher-than-normal BMI growth pattern.” Details on calculating individual risk probabilities and use of individual risk score calculations along with clinical case scenarios are provided in the Text S3 (Additional file 1). BMI, body mass index; BF, breastfeeding; GDM, gestational diabetes; GWG, gestational weight gain; m, month(s); LGA, large-for-gestational-age; SES, socioeconomic status; SGA, small-for-gestational-age; y, year(s).

## Data Availability

The datasets analyzed during the current study are not publicly available because participants did not explicitly consent to the sharing of their data as per European Union’s General Data Protection Regulation and the corresponding German privacy laws. However, data are available from the corresponding author (Regina Ensenauer, principal investigator) on reasonable request for researchers who meet the criteria for access to confidential data.

## Abbreviations

AGA: Average-for-gestational-age
AUROC: Area under the receiver operating characteristic
BMI: Body mass index
CI: Confidence interval
GCT: Glucose challenge test
GDM: Gestational diabetes
GWG: Gestational weight gain
IADPSG: International Association of Diabetes and Pregnancy Study Groups
IQR: Interquartile range
KiGGS: German Health Interview and Examination Survey for Children and Adolescents
LASSO: Least absolute shrinkage and selection operator
LGA: Large-for-gestational-age
OGTT: Oral glucose tolerance test
OR: Odds ratio
PEACHES: Programming of Enhanced Adiposity Risk in CHildhood–Early Screening
PEPO: PErinatal Prevention of Obesity
SES: Socioeconomic status
SGA: Small-for-gestational-age
T1D: Type 1 diabetes
T2D: Type 2 diabetes
WHO: World Health Organization
