# Supplementary material for: Predicting the earliest deviation in weight gain in the course towards manifest overweight in offspring exposed to obesity in pregnancy: a longitudinal cohort study
Source: BMC Med. 2022 Apr 14;20:156. doi: 10.1186/s12916-022-02318-z (PMC9008920; doi:10.1186/s12916-022-02318-z)
Supplement: Supplementary file 1 — Additional file 1: S1 STROBE Checklist. S2 TRIPOD Statement. Text S1. Statistical analysis plan. Text S2. Statistical methods. Text S3. Quantification of individual risk. Figure S1. Influence of maternal obesity on offspring BMI growth outcomes. Shown are ORs and 95% CIs of the influence of maternal pre-conception obesity on BMI growth outcomes up to age 5 years in all offspring belonging to upper BMI growth clusters from the PEACHES cohort study. Values were derived from univariate logistic regression. aThe term “multiple occasions” was defined as having BMI z-scores >1 SD [51] at least 5 out of 6 times at the well-child visits at age 6 months, 1 year, 2 years, 3 years, 4 years, and 5 years. BMI, body mass index; CI, confidence interval; OR, odds ratio; PEACHES, Programming of Enhanced Adiposity Risk in CHildhood–Early Screening. Figure S2. Proportion of offspring in upper and lower BMI growth clusters according to birth weight category. Shown are percentages in offspring of mothers with obesity (panel A) and without (panel B) enrolled in the PEACHES cohort study, according to their birth weight category for gestational age and sex. AGA, average-for-gestational-age; BMI, body mass index; LGA, large-for-gestational-age; PEACHES, Programming of Enhanced Adiposity Risk in CHildhood–Early Screening; SGA, small-for-gestational-age. Figure S3. Mean BMI growth clusters by birth weight category in offspring of mothers with and without obesity. Shown are mean BMI z-score growth clusters from birth to age 6 months (panel A, C) and birth to age 5 years (panel B, D) by birth weight category for gestational age and sex in offspring of mothers with and without obesity enrolled in the PEACHES cohort study. AGA, average-for-gestational-age; BMI, body mass index; LGA, large-for-gestational-age; PEACHES, Programming of Enhanced Adiposity Risk in CHildhood–Early Screening; SGA, small-for-gestational-age. Figure S4. Effects of prenatal and postnatal factors on BMI growth outcomes in o [file 12916_2022_2318_MOESM1_ESM.docx]

**Additional file 1.**

Gomes D, Le L, Perschbacher S, et al. Predicting the earliest deviation in weight gain in the course towards manifest overweight in offspring exposed to obesity in pregnancy: a longitudinal cohort study

**Table of Contents**

S1 STROBE Checklist.

S2 TRIPOD Statement.

Text S1. Statistical analysis plan.

Text S2. Statistical methods.

Text S3. Quantification of individual risk.

Figure S1. Influence of maternal obesity on offspring BMI growth outcomes.

Figure S2. Proportion of offspring in upper and lower BMI growth clusters according to birth weight category.

# Figure S3. Mean BMI growth clusters by birth weight category in offspring of mothers with and without obesity.

# Figure S4. Effects of prenatal and postnatal factors on BMI growth outcomes in offspring of mothers without obesity.

Figure S5. Calibration plots of prediction models for identifying a “higher-than-normal BMI growth pattern” in the validation cohort.

Table S1. Offspring follow-up rates in the study populations.

Table S2. Mean BMI z-scores by BMI growth cluster in offspring of mothers with and without obesity.

Table S3. Offspring BMI growth dynamics in consecutive life phases after birth following exposure to gestational obesity.

Table S4. Predictive performance of a sequential algorithm to identify higher-than-normal BMI growth in offspring of mothers without obesity.

Table S5. Scoring system for quantification of risk of higher-than-normal BMI growth in young offspring.

**Abbreviations:** AGA, average-for-gestational-age; AUROC, area under the receiver operating characteristic; BF, breastfeeding; BMI, body mass index; CI, confidence interval; exp, exponential function; GDM, gestational diabetes; GWG, gestational weight gain; LASSO, least absolute shrinkage and selection operator; LGA, large-for-gestational-age; OR, odds ratio; PEACHES, Programming of Enhanced Adiposity Risk in Childhood–Early Screening; PEPO, PErinatal Prevention of Obesity; ROC, receiver operating characteristic; SES, socioeconomic status; SGA, small-for-gestational-age; T1D, type 1 diabetes; T2D, type 2 diabetes; WHO, World Health Organization.

**S1 STROBE Checklist.**

STROBE Statement—Checklist of items that should be included in reports of ***cohort studies***

|  | Item No | Recommendation | Author’s Response |
| --- | --- | --- | --- |
| **Title and abstract** | 1 | (*a*) Indicate the study’s design with a commonly used term in the title or the abstract | Pages 1 and 3 to 4, Title; Abstract, “Methods”. |
|  |  | (*b*) Provide in the abstract an informative and balanced summary of what was done and what was found | Pages 3 to 4, Abstract, “Background”, “Methods”, and “Results”. |
| Introduction | | |  |
| Background/rationale | 2 | Explain the scientific background and rationale for the investigation being reported | Pages 5 to 6, Introduction, paragraphs 1 to 3. |
| Objectives | 3 | State specific objectives, including any prespecified hypotheses | Page 6, Introduction, paragraph 4. |
| Methods | | |  |
| Study design | 4 | Present key elements of study design early in the paper | Page 7, Methods, “Study design and populations”. |
| Setting | 5 | Describe the setting, locations, and relevant dates, including periods of recruitment, exposure, follow-up, and data collection | Pages 7 to 13, Methods, “Study design and populations”, “Procedures”; Fig 1. |
| Participants | 6 | (*a*) Give the eligibility criteria, and the sources and methods of selection of participants. Describe methods of follow-up | Pages 7 to 8, Methods, “Study design and populations” and “Procedures”: “Inclusion criteria for analysis”, Pages 12 to 13, “Growth outcomes until age 5 years”; Fig 1; Additional file 1, Table S1. |
|  |  | (*b*) For matched studies, give matching criteria and number of exposed and unexposed | Not applicable. |
| Variables | 7 | Clearly define all outcomes, exposures, predictors, potential confounders, and effect modifiers. Give diagnostic criteria, if applicable | Pages 8 to 13, Methods, “Potential predictors of higher-than-normal BMI growth”, “Growth outcomes until age 5 years”. |
| Data sources/ measurement | 8* | For each variable of interest, give sources of data and details of methods of assessment (measurement). Describe comparability of assessment methods if there is more than one group | Pages 8 to 13, Methods, “Potential predictors of higher-than-normal BMI growth”, “Growth outcomes until age 5 years”. |
| Bias | 9 | Describe any efforts to address potential sources of bias | Pages 7 to 8, Methods, “Study design and populations”; Pages 8 to 9, “Potential predictors of higher-than-normal BMI growth”, Page 14, “Statistical analysis”; Page 34, Discussion, paragraph 7; Additional file 1, Table S1. |
| Study size | 10 | Explain how the study size was arrived at | Page 8, Methods, “Inclusion criteria for analysis”, Page 14, “Statistical analysis”; Fig 1; Additional file 1, Text S2. “Statistical methods.” (Page 13), Table S1. |
| Quantitative variables | 11 | Explain how quantitative variables were handled in the analyses. If applicable, describe which groupings were chosen and why | Pages 8 to 13, Methods, “Inclusion criteria for analysis”, “Potential predictors of higher-than-normal BMI growth”, “Growth outcomes until age 5 years”; Page 19 to 21, Results, “BMI growth patterns in offspring”. |
| Statistical methods | 12 | (*a*) Describe all statistical methods, including those used to control for confounding | Pages 14 to 15, Methods, “Statistical analysis”. Additional file 1, Text S1. “Statistical analysis plan.”, Text S2. “Statistical methods.”, Text S3. “Quantification of individual risk.” |
|  |  | (*b*) Describe any methods used to examine subgroups and interactions | Page 14, Methods, “Statistical analysis”; Additional file 1, Text S1. “Statistical analysis plan.”, Text S2. “Statistical methods.” |
|  |  | (*c*) Explain how missing data were addressed | Page 14, Methods, “Statistical analysis”; Table 1 (footnote); Fig 1; Additional file 1, Text S2. “Statistical methods.”, paragraph 1.6 (Page 14), Table S1 and Table S2. |
|  |  | (*d*) If applicable, explain how loss to follow-up was addressed | Any losses to follow-up were excluded from analysis.  Page 14, Methods, “Statistical analysis”; Table 1 (footnote); Fig 1; Additional file 1, Table S1 and Table S2. |
|  |  | (*e*) Describe any sensitivity analyses | We used an independent cohort PEPO for validation purposes and to assess the robustness of our results using data-driven approaches. We conducted analyses in two different groups: offspring of mothers with obesity and offspring of mothers without obesity.  Page 7, Methods, “Study design and populations”, Pages 14 to 15, “Statistical analysis”; Results, “BMI growth patterns in offspring”, “Sequential prediction of higher-than-normal BMI growth”; Table 2; Additional file 1, Text S1. “Statistical analysis plan.”, Text S2. “Statistical methods.”, Fig S1, Table S4. |
| Results | | |  |
| Participants | 13* | (a) Report numbers of individuals at each stage of study—eg numbers potentially eligible, examined for eligibility, confirmed eligible, included in the study, completing follow-up, and analysed | Pages 15 to 17, Results, “Characteristics of study populations”, Page 19, “BMI growth patterns in offspring”; Table 1; Fig 1; Additional file 1, Tables S1 to S4, Fig. S2. |
|  |  | (b) Give reasons for non-participation at each stage | Page 14, Methods, “Statistical analysis”; Pages 15 to 16, Results, “Characteristics of study populations”; Fig 1; Additional file 1, Tables S1 and S2. |
|  |  | (c) Consider use of a flow diagram | Fig 1. |
| Descriptive data | 14* | (a) Give characteristics of study participants (eg demographic, clinical, social) and information on exposures and potential confounders | Pages 15 to 17, Results, “Characteristics of study populations”; Table 1. |
|  |  | (b) Indicate number of participants with missing data for each variable of interest | Number of participants with complete data are given in the following figures and tables:  Fig 1; Tables 1 and 2; Additional file 1, Tables S1 to S4, Fig. S2.  Participants with any missing data were excluded from analysis. |
|  |  | (c) Summarise follow-up time (eg, average and total amount) | Pages 12 to 13, Methods, “Growth outcomes until age 5 years”; Table 1; Additional file 1, Tables S1 and S2. |
| Outcome data | 15* | Report numbers of outcome events or summary measures over time | Pages 19 to 22, Results, “BMI growth patterns in offspring”, “Higher-than-normal BMI growth patterns in consecutive early-life phases”; Figs 1 and 2; Table 2; Additional file 1, Tables S1 to S4. |
| Main results | 16 | (*a*) Give unadjusted estimates and, if applicable, confounder-adjusted estimates and their precision (eg, 95% confidence interval). Make clear which confounders were adjusted for and why they were included. | Page 9, Methods, “Potential predictors of higher-than-normal BMI growth”; Pages 22 to 27, Results, “Risk factors of higher-than-normal BMI growth”, “Sequential prediction of higher-than-normal BMI growth”; Fig 3 (including legend); Table 2; Additional file 1, Fig S4 (including legend), Table S4. |
|  |  | (*b*) Report category boundaries when continuous variables were categorized | Pages 10 to 13, Methods, “Potential predictors of higher-than-normal BMI growth”, “Growth outcomes until age 5 years”; Pages 19 to 22, Results, “BMI growth patterns in offspring”, “Higher-than-normal BMI growth patterns in consecutive early-life phases”; Additional file 1, Fig S1, Table S3. |
|  |  | (*c*) If relevant, consider translating estimates of relative risk into absolute risk for a meaningful time period | Not applicable. |
| Other analyses | 17 | Report other analyses done—eg analyses of subgroups and interactions, and sensitivity analyses | Not applicable. |
| Discussion | | |  |
| Key results | 18 | Summarise key results with reference to study objectives | Page 28, Discussion, paragraph 1. |
| Limitations | 19 | Discuss limitations of the study, taking into account sources of potential bias or imprecision. Discuss both direction and magnitude of any potential bias | Pages 34 to 35, Discussion, paragraph 8. |
| Interpretation | 20 | Give a cautious overall interpretation of results considering objectives, limitations, multiplicity of analyses, results from similar studies, and other relevant evidence | Pages 28 to 35, Discussion, paragraphs 2 to 8. |
| Generalisability | 21 | Discuss the generalisability (external validity) of the study results | Page 34, Discussion, paragraphs 7 and 8. |
| Other information | | |  |
| Funding | 22 | Give the source of funding and the role of the funders for the present study and, if applicable, for the original study on which the present article is based | Pages 36 to 37. |

*Give information separately for exposed and unexposed groups.

**Note:** An Explanation and Elaboration article discusses each checklist item and gives methodological background and published examples of transparent reporting. The STROBE checklist is best used in conjunction with this article (freely available on the Web sites of PLoS Medicine at http://www.plosmedicine.org/, Annals of Internal Medicine at http://www.annals.org/, and Epidemiology at http://www.epidem.com/). Information on the STROBE Initiative is available at http://www.strobe-statement.org.

**S2 TRIPOD Statement.**

| **TRIPOD Checklist: Prediction Model Development and Validation** | | | | |
| --- | --- | --- | --- | --- |
| **Section/Topic** | **Item*** |  | **Checklist Item** | **Page** |
| **Title and abstract** | | | | |
| Title | 1 | D;V | Identify the study as developing and/or validating a multivariable prediction model, the target population, and the outcome to be predicted. | Page 1, Title. |
| Abstract | 2 | D;V | Provide a summary of objectives, study design, setting, participants, sample size, predictors, outcome, statistical analysis, results, and conclusions. | Pages 3 to 4, Abstract. |
| **Introduction** | | | | |
| Background and objectives | 3a | D;V | Explain the medical context (including whether diagnostic or prognostic) and rationale for developing or validating the multivariable prediction model, including references to existing models. | Pages 5 to 6, Background, paragraphs 1 to 3. |
|  | 3b | D;V | Specify the objectives, including whether the study describes the development or validation of the model or both. | Page 6, Background, paragraph 4. |
| **Methods** | | | | |
| Source of data | 4a | D;V | Describe the study design or source of data (e.g., randomized trial, cohort, or registry data), separately for the development and validation datasets, if applicable. | Page 7, Methods, “Study design and populations”. |
|  | 4b | D;V | Specify the key study dates, including start of accrual; end of accrual; and, if applicable, end of follow-up. | Page 7, Methods, “Study design and populations”, Pages 12 to 13, “Growth outcomes until age 5 years”. |
| Participants | 5a | D;V | Specify key elements of the study setting (e.g., primary care, secondary care, general population) including number and location of centres. | Page 7, Methods, “Study design and populations”. |
|  | 5b | D;V | Describe eligibility criteria for participants. | Page 7, Methods, “Study design and populations”, Page 8, “Inclusion criteria for analysis”. |
|  | 5c | D;V | Give details of treatments received, if relevant. | Not applicable. |
| Outcome | 6a | D;V | Clearly define the outcome that is predicted by the prediction model, including how and when assessed. | Page 13, Methods, “Growth outcomes until age 5 years” (paragraph 4). |
|  | 6b | D;V | Report any actions to blind assessment of the outcome to be predicted. | Not applicable. |
| Predictors | 7a | D;V | Clearly define all predictors used in developing or validating the multivariable prediction model, including how and when they were measured. | Pages 8 to 12, Methods, “Potential predictors of higher-than-normal BMI growth”. |
|  | 7b | D;V | Report any actions to blind assessment of predictors for the outcome and other predictors. | Not applicable. |
| Sample size | 8 | D;V | Explain how the study size was arrived at. | Page 8, Methods, “Inclusion criteria for analysis”, Page 14 “Statistical analysis”; Fig 1; Additional file 1, Text S2. “Statistical methods.” (Page 13), Table S1. |
| Missing data | 9 | D;V | Describe how missing data were handled (e.g., complete-case analysis, single imputation, multiple imputation) with details of any imputation method. | Page 14, Methods, “Statistical analysis”; Table 1 (footnote); Fig 1; Additional file 1, Text S2. “Statistical methods.”, paragraph 1.6 (Page 14), Table S1 and Table S2. |
| Statistical analysis methods | 10a | D | Describe how predictors were handled in the analyses. | Page 14, Methods, “Statistical analysis”; Additional file 1, Text S1. “Statistical analysis plan.”,  Text S2. “Statistical methods.”. |
|  | 10b | D | Specify type of model, all model-building procedures (including any predictor selection), and method for internal validation. | Page 14, Methods, “Statistical analysis”; Additional file 1, Text S1. “Statistical analysis plan.”, Text S2. “Statistical methods.”. |
|  | 10c | V | For validation, describe how the predictions were calculated. | Additional file 1, Text S1. “Statistical analysis plan.”,  Text S2. “Statistical methods.”, Table S4; Table 2. |
|  | 10d | D;V | Specify all measures used to assess model performance and, if relevant, to compare multiple models. | Table 2; Additional file 1, Text S2. “Statistical methods.”, Table S4. |
|  | 10e | V | Describe any model updating (e.g., recalibration) arising from the validation, if done. | Not applicable. |
| Risk groups | 11 | D;V | Provide details on how risk groups were created, if done. | Page 14, Methods, “Statistical analysis”. |
| Development vs. validation | 12 | V | For validation, identify any differences from the development data in setting, eligibility criteria, outcome, and predictors. | Pages 7 to 15, Methods, “Study design and populations”, “Procedures”, “Statistical analysis”; Pages 15 to 17, Results, “Characteristics of study populations”; Table 1; Additional file 1, Table S1. |
| **Results** | | | | |
| Participants | 13a | D;V | Describe the flow of participants through the study, including the number of participants with and without the outcome and, if applicable, a summary of the follow-up time. A diagram may be helpful. | Fig. 1.  Additional file 1, Table S3. |
|  | 13b | D;V | Describe the characteristics of the participants (basic demographics, clinical features, available predictors), including the number of participants with missing data for predictors and outcome. | Pages 15 to 17, Results, “Characteristics of study populations”; Tables 1 and 2; Additional file 1, Tables S1 to S4. |
|  | 13c | V | For validation, show a comparison with the development data of the distribution of important variables (demographics, predictors and outcome). | Pages 15 to 17, Results, “Characteristics of study populations”; Table 1. |
| Model development | 14a | D | Specify the number of participants and outcome events in each analysis. | Number of participants with complete data are given in the following figures and tables:  Fig 1; Tables 1 and 2; Additional file 1, Tables S1 to S4. |
|  | 14b | D | If done, report the unadjusted association between each candidate predictor and outcome. | Not applicable. |
| Model specification | 15a | D | Present the full prediction model to allow predictions for individuals (i.e., all regression coefficients, and model intercept or baseline survival at a given time point). | Additional file 1, Table S5. |
|  | 15b | D | Explain how to use the prediction model. | Additional file 1, Text S3. “Quantification of individual risk.”. |
| Model performance | 16 | D;V | Report performance measures (with CIs) for the prediction model. | Table 2;  Additional file 1, Table S4. |
| Model-updating | 17 | V | If done, report the results from any model updating (i.e., model specification, model performance). | Not applicable. |
| **Discussion** | | | | |
| Limitations | 18 | D;V | Discuss any limitations of the study (such as nonrepresentative sample, few events per predictor, missing data). | Pages 34 to 35, Discussion. |
| Interpretation | 19a | V | For validation, discuss the results with reference to performance in the development data, and any other validation data. | Page 34, Discussion. |
|  | 19b | D;V | Give an overall interpretation of the results, considering objectives, limitations, results from similar studies, and other relevant evidence. | Pages 28 to 35, Discussion. |
| Implications | 20 | D;V | Discuss the potential clinical use of the model and implications for future research. | Page 35, Discussion. |
| **Other information** | | | | |
| Supplementary information | 21 | D;V | Provide information about the availability of supplementary resources, such as study protocol, Web calculator, and datasets. | Additional file 1, Text S1. “Statistical analysis plan.”, Text S3. “Quantification of individual risk.”, Table S5. |
| Funding | 22 | D;V | Give the source of funding and the role of the funders for the present study. | Pages 36 to 37. |

*Items relevant only to the development of a prediction model are denoted by D, items relating solely to a validation of a prediction model are denoted by V, and items relating to both are denoted D;V. We recommend using the TRIPOD Checklist in conjunction with the TRIPOD Explanation and Elaboration document.

**Text S1. Statistical analysis plan.**

**Manuscript: Predicting the earliest deviation in weight gain in the course towards manifest overweight in offspring exposed to obesity in pregnancy: a longitudinal cohort study**

Ulrich Mansmann, Delphina Gomes, Regina Ensenauer, and Lien Le

Munich, 2020-01-13

**Contents**

[1 Study population 9](#_Toc59986018)

[2 Calculation of specific predictors and BMI outcome variables 9](#_Toc59986019)

[3 Statistical analyses 10](#_Toc59986020)

# **Study population**

The following inclusion and exclusion criteria will be applied for the analyses in the children enrolled in the Programming of Enhanced Adiposity Risk in Childhood–Early Screening (PEACHES) cohort and the PErinatal Prevention of Obesity (PEPO) cohort (validation):

Inclusion criteria:

1. Mothers with or without pre-conception obesity
2. Singleton pregnancy
3. Absence of type 1 diabetes (T1D) or type 2 diabetes (T2D) in mothers
4. Full-term (≥37 weeks 0 days of gestation) live birth

Exclusion criteria:

1. Underweight mothers
2. Twin/multiple pregnancy
3. Presence of T1D or T2D in mothers
4. Preterm children (gestational age ≤36 weeks 6 days of gestation)

# **Calculation of specific predictors of higher-than-normal BMI growth in offspring**

The majority of predictors including maternal pre-conception body mass index (BMI), gestational diabetes (GDM), parity, smoking during pregnancy, sex, socioeconomic status (SES), and breastfeeding status at 1, 3, and 6 months will be extracted from the PEACHES and PEPO databases. Gestational weight gain (GWG) and birth weight categories will be calculated.

- 1. **Calculation of total GWG**

Total GWG in kilograms will be calculated as the diﬀerence between the last measured weight before delivery and pre-conception weight and will be classified as inadequate, adequate, or excessive according to the BMI-specific recommendations of the Institute of Medicine (now known as the National Academy of Medicine)/National Research Council [38]. Pre-conception weight will be based on data measured at the first antenatal visit if the visit was before 12 weeks 6 days of gestation or on reported and documented data abstracted from the pregnancy record booklet if the first visit was later than the 13^th^ week of gestation.

- 1. **Calculation of birth weight categories for gestational age and sex**

We will group offspring according to their birth weight adjusted for gestational age and sex into large-for-gestational-age (LGA, >90th percentile), average-for-gestational-age (AGA, 10th to 90th percentile), or small-for-gestational-age (SGA, <10th percentile) categories. These cut-oﬀs were based on the German reference population [46].

- 1. **Calculation of offspring BMI z-scores**

Offspring BMI z-scores will be calculated according to World Health Organization (WHO) age- and sex-specific growth standards [51].

- 1. **Calculation of frequencies of BMI z-score >1 SD**

“Early phase” and “late phase” of oﬀspring BMI growth will be defined as the period between 6 months to 2 years and 3 years to 5 years, respectively. Children will be classified as having a “higher-than-normal BMI growth pattern” (BMI z-score >1 SD [51] at least twice) within each growth phase.

Among all children belonging to the group of high BMI growth, we will also categorize oﬀspring with repeated occasions of BMI z-score >1 SD at the 6-month, 1-year, 2-year, 3-year, 4-year, and 5-year follow-up visits to identify oﬀspring with the highest risk of developing preschool overweight.

# **Statistical analyses**

- 1. **Longitudinal cluster analysis**

Analysis with k-means clustering will aim to divide children into BMI growth clusters, which will be characterized by distinct BMI development patterns from birth to 5 years of age. The cluster analysis will be done for children of mothers with and without pre-conception obesity, respectively.

- 1. **Validation analysis of cluster findings**

The BMI growth clusters that will be identified in offspring enrolled in the PEACHES cohort will be externally validated in the PEPO cohort. The validation procedure will first classify offspring into different clusters using random forests. Next, we will apply this cluster-derived classification rule to the children in the PEPO cohort: offspring will be categorized into one of the different cluster-derived classes. Next, we will quantify whether different cluster classes can discriminate the BMI z-scores at age 5 years of the PEPO children. To this end, the receiver operating characteristic (ROC) will be determined and the area under the ROC (AUROC) will be calculated to quantify the discriminatory ability of the cluster-derived classification rule.

- 1. **Analysis of risks of adverse BMI growth outcomes**

Using logistic regression analyses, we will calculate odds ratios for the following:

1. Outcome = child overweight (including obesity), predictor = BMI growth cluster, subgroups = mothers with and without obesity. To evaluate the influence of upper cluster BMI growth on the manifestation of childhood overweight/obesity, we will perform regression analysis with oﬀspring overweight/obesity (yes versus no) as outcome and cluster of BMI growth (upper versus lower) as influencing factor in oﬀspring subgroups of mothers with and without obesity. This analysis will be performed for both offspring age 4 and 5 years, respectively.
2. Outcome = child overweight (including obesity), predictor = maternal pre-conception obesity, subgroup = all oﬀspring in upper BMI growth clusters. To evaluate the influence of maternal pre-conception obesity on overweight/obesity risk in all oﬀspring with an upper BMI growth trajectory, we will conduct regression analysis with oﬀspring overweight/obesity (yes versus no) as outcome and maternal pre-conception BMI group (with obesity versus without) as influencing factor in the population of oﬀspring growing in the upper BMI growth clusters (of both mothers with and without obesity). This analysis will be performed for both offspring age 4 and 5 years, respectively.
3. Outcome = at least 5 out of 6 occurrences of having a BMI z-score >1 SD, predictor = maternal pre-conception obesity, subgroup = all oﬀspring in upper BMI growth clusters. Within the oﬀspring population growing in the upper BMI clusters, we will calculate the odds for at least 5 out of 6 occurrences of having a BMI z-score >1 SD with number of occurrences (≥5 versus ≤4) as outcome and maternal pre-conception BMI group (with obesity versus without) as influencing factor.
   1. **Analysis of the influence of pre- and postnatal factors on higher-than-normal BMI growth**

We will explore the simultaneous eﬀects of prenatal and postnatal factors on upper BMI growth clusters and “higher-than-normal BMI growth pattern” during the early phase and the late phase in oﬀspring by logistic regression using backward selection. These analyses will be conducted separately for oﬀspring of mothers with and without obesity. The following prenatal and postnatal predictors will be included:

1. Outcome = upper BMI growth cluster, potential predictors = maternal pre-conception BMI group, total GWG, GDM, parity, smoking during pregnancy, sex, birth weight categories for gestational age and sex, SES, and breastfeeding status at 1 month.
2. Outcome = “higher-than-normal BMI growth pattern” in early phase, potential predictors = maternal pre-conception BMI group, total GWG, GDM, parity, smoking during pregnancy, sex, birth weight categories for gestational age and sex, SES, and breastfeeding status at 1 month.
3. Outcome = “higher-than-normal BMI growth pattern” in late phase, potential predictors = maternal pre-conception BMI group, total GWG, GDM, parity, smoking during pregnancy, sex, birth weight categories for gestational age and sex, SES, breastfeeding status at 1 month, and “higher-than-normal BMI growth pattern” in early phase.
   1. **Prediction analysis**

Using penalized least absolute shrinkage and selection operator (LASSO) regression analysis, we will explore the predictive power of prenatal and postnatal factors including current child BMI status to predict “higher-than-normal BMI growth pattern” in early and late phases of growth at ages 3 months, 1 year, and 2 years in oﬀspring. LASSO regression analysis will be conducted for early phase and late phase separately in offspring groups of mothers with and without obesity.

All prediction models will include the following prenatal and postnatal factors and their two-fold interactions. Prenatal/postnatal factors included in prediction models are as follows:

1. Prediction at age 3 months = maternal pre-conception BMI group, total GWG, GDM, parity, smoking during pregnancy, sex, birth weight categories for gestational age and sex, SES, breastfeeding status at 1 month, breastfeeding status at 3 months, and BMI z-score >1 SD at age 3 months.
2. Prediction at age 1 year = maternal pre-conception BMI group, total GWG, GDM, parity, smoking during pregnancy, sex, birth weight categories for gestational age and sex, SES, breastfeeding status at 1 month, breastfeeding status at 3 months, breastfeeding status at 6 months, and BMI z-score >1 SD at age 1 year.
3. Prediction at age 2 years = maternal pre-conception BMI group, total GWG, GDM, parity, smoking during pregnancy, sex, birth weight categories for gestational age and sex, SES, breastfeeding status at 1 month, breastfeeding status at 3 months, breastfeeding status at 6 months, and BMI z-score >1 SD at age 2 years.

Internal validation will be performed with the whole PEACHES dataset. AUROC will be calculated to assess the internal prediction performance of the selected model from the LASSO regression analysis. Prediction models at ages 1 year and 2 years will be validated using available data of the PEPO cohort to assess the external prediction performance.

We will calculate different cut-oﬀs and determine cut-off points that are:

1. closest to the upper left corner of the ROC curve, where equal weight is given to false negative and false positive predictions,
2. closest to the upper left corner of the ROC curve, where double weight is given to false positive predictions,
3. closest to the upper left corner of the ROC curve, where double weight is given to false negative predictions,
4. 90th percentile of the linear predictors of children without “higher-than-normal BMI growth pattern”.

**Text S2. Statistical methods.**

The current analysis is exploratory and hypothesis-generating (discovery) on the data of the Programming of Enhanced Adiposity Risk in Childhood–Early Screening (PEACHES) cohort. No formal sample size calculation was done, and no primary hypothesis was formulated. Independent external validation of analyses was performed on the data of the PErinatal Prevention of Obesity (PEPO) cohort. The sample size calculation for the validation was based on example 8.3 (page 226) of Pepe (2003) [53]. A total of 500 children (assuming a 20% prevalence of children with higher-than-normal body mass index [BMI] growth) were needed to reject the null hypothesis (area under the receiver operating characteristic [AUROC] ≤0.55) given the alternative (AUROC=0.70) on a 5% level with a power of at least 90%.

- 1. **Assessment of the agreement between self-reported pre-conception weight and weight measured at the first antenatal visit**

We assessed the agreement between maternal pre-conception weight self-reported and weight measured at the first antenatal visit in the PEACHES data using the principles of the Bland-Altman method [37]. We further estimated the correlation between the two maternal weight measurements using the Pearson product-moment correlation.

- 1. **Cluster analysis and assessment of adequate BMI growth clusters**

Cluster analysis was performed using the k-means technique in children of the PEACHES cohort. K-means clustering is a non-parametric approach aimed to group children’s growth trajectories into clusters characterized by distinct weight development patterns from birth to 5 years of age. The best number of clusters was chosen by applying the Calinski & Harabasz criterion, where maximum variances between clusters and minimum variances within clusters are reached [54]. The criterion gave two options: setting the number of clusters to two or three.

For the analyses, we used the three-cluster option. The two lower clusters were combined and compared to the upper cluster. We applied the least absolute shrinkage and selection operator (LASSO) logistic regression and 10-fold cross validation to identify models that can best divide the offspring into separate BMI growth clusters.

- 1. **Validation of BMI growth clusters**

Since the number of offspring BMI z-score measurements differed between the PEACHES (maximum of 9) and the PEPO (maximum of 4) cohorts, clusters of BMI growth were validated in the PEPO cohort based on random forests. The BMI growth clusters obtained from offspring enrolled in the PEACHES cohort were validated by the following steps.

In the first step, using PEACHES data from birth to age 3 months, offspring were classified into upper or lower BMI growth clusters. In the second step, the children of the PEPO cohort were stratified into two groups (i.e. into a potential lower versus a potential upper BMI growth cluster) according to the classification rule of step one. In the third step, within the PEPO cohort, the AUROC was calculated based on the BMI growth cluster membership determined in step two and the BMI z-scores at age 5 years. These steps allowed assessing, within the PEPO cohort, whether the BMI growth cluster membership discriminated children based on their BMI z-score value at age 5 years, in the respective groups of children of mothers with and without obesity.

- 1. **Risks of adverse BMI growth outcomes**

Based on univariate logistic regression analysis, we calculated the risks of several adverse BMI growth outcomes including i) multiple occasions (≥5 occurrences) of offspring BMI z-score >1 SD between age 6 months to 5 years and ii) child overweight (including obesity) at both age 4 and 5 years, respectively.

Firstly, we evaluated the influence of growing in the upper cluster of BMI growth on the manifestation of childhood overweight/obesity in the offspring populations of mothers with or without obesity. We used the presence of childhood overweight/obesity (yes versus no) as outcome and cluster membership of BMI growth (upper versus lower) as influencing factor.

Next, we studied the influence of maternal pre-conception obesity on the manifestation of childhood overweight/obesity in all offspring with upper BMI growth trajectory (Figure S1). We used the presence of childhood overweight/obesity (yes versus no) as outcome and the maternal pre-conception BMI group (mothers with obesity versus without) as influencing factor.

Among all children belonging to the upper clusters of BMI growth, we classified offspring BMI z-score as below, equal or above 1 SD [51] at each of the well-child visits at age 6 months, 1 year, 2 years, 3 years, 4 years, and 5 years. To identify offspring with the highest risk of developing preschool overweight, we calculated the odds for having a BMI z-score >1 SD on at least 5 out of 6 occasions in offspring growing in the upper BMI growth clusters (Figure S1). We used the number of occurrences (≥5 versus ≤4) as outcome and the maternal pre-conception BMI group (mothers with obesity versus without) as influencing factor.

- 1. **Identification of prenatal and postnatal risk factors of higher-than-normal BMI growth**

Using log-linear models, we compared the structural differences related to a “higher-than-normal BMI growth pattern” during the early phase and the late phase in offspring of mothers with obesity versus without.

A series of multivariable logistic regression analyses were performed to identify risk factors related to i) the cluster of upper BMI growth membership from birth to 5 years and ii) the main outcome of a “higher-than-normal BMI growth pattern” during the early phase and the late phase of growth, respectively, in offspring of mothers with or without obesity. For models assessing the effect of prenatal and postnatal factors on higher-than-normal BMI growth in the late phase, we used “higher-than-normal BMI growth pattern” in the early phase as an additional potentially influencing variable.

After starting with a comprehensive model that included all potential prenatal and postnatal factors, use of a backward selection method resulted in combinations of factors influencing higher-than-normal BMI growth of offspring during the entire first 5 years after birth and during the early phase and the late phase within this period, respectively. A prenatal or postnatal risk factor was considered relevant if the 95% confidence interval (CI) of its odds ratio did not contain 1.

- 1. **Development and validation of risk prediction models**

Using the dataset of the PEACHES cohort, we developed several risk models to predict a “higher-than-normal BMI growth pattern” in offspring during their early and late phases of growth, respectively. For the prediction analyses, we only included children with complete data on prenatal and postnatal factors and longitudinal anthropometric measurements. Missing values were not imputed since existing proposals for multiple imputation in penalized logistic regression models (see e.g. R-package MAMI [55] [<http://mami.r-forge.r-project.org/>]) have not been validated up to now.

Consecutive prediction models were developed using robust techniques such as LASSO, which enabled both individual risk scoring and risk probability assessment at the well-child visits at ages 3 months, 1 year, and 2 years. For each prediction model, we calculated several cut-off score values, because there are no standard criteria for the identification of risk thresholds to predict childhood excess BMI growth [56]. We calculated the sensitivity, specificity, positive and negative predictive values, and likelihood ratios, with corresponding 95% CIs for the prediction models in both the discovery and validation cohorts. Positive likelihood ratio values of 2, 3, and 4 indicate an increase in the likelihood of a “higher-than-normal BMI growth pattern” in offspring identified as “at risk” with a probability of 15%, 20%, and 25%, respectively [57]. Negative likelihood ratio values of 0.5, 0.4, and 0.3 indicate a decrease in the likelihood of a “higher-than-normal BMI growth pattern” in offspring identified as “not at risk” with a probability of 15%, 20%, and 25%, respectively [57].

Prediction models included the following prenatal and postnatal factors (including child’s BMI status at the respective prediction time point) and interactions between factors:

**Prediction of higher-than-normal BMI growth in early phase at age 3 months:** maternal pre-conception BMI group, total gestational weight gain (GWG), gestational diabetes (GDM), parity, smoking during pregnancy, sex, birth weight category for gestational age and sex, socioeconomic status (SES), breastfeeding status at 1 month, breastfeeding status at 3 months, and BMI z-score >1 SD at age 3 months.

**Prediction of higher-than-normal BMI growth in late phase at age 1 year:** maternal pre-conception BMI group, total GWG, GDM, parity, smoking during pregnancy, sex, birth weight category for gestational age and sex, SES, breastfeeding status at 1 month, breastfeeding status at 3 months, breastfeeding status at 6 months, and BMI z-score >1 SD at age 1 year.

**Prediction of higher-than-normal BMI growth in late phase at age 2 years:** maternal pre-conception BMI group, total GWG, GDM, parity, smoking during pregnancy, sex, birth weight category for gestational age and sex, SES, breastfeeding status at 1 month, breastfeeding status at 3 months, breastfeeding status at 6 months, and BMI z-score >1 SD at age 2 years.

Prediction models at offspring ages 1 and 2 years were externally validated by applying them to corresponding mother-child pairs of the PEPO cohort. We were unable to externally validate the prediction model at age 3 months because the PEPO cohort does not offer data on offspring BMI z-scores at age 3 months. We generated calibration plots and provided calibration slopes and intercepts to assess the agreement between the observed and predicted probabilities of the outcome using the validation cohort PEPO [58, 59]. Calibration was considered as optimal, if the observed and predicted risks were on a 45-degree diagonal line.

All P values presented were two-sided. We used the statistical software package R version 3.5.1 [60] supported by the following version-specific packages: kml [61] for the cluster analysis, MASS [62] for backward selection in association analyses, glmnet [63] for LASSO regression analysis, lme4 [64] for analyses using mixed models, and ROCR [65] for prediction analyses.

**Text S3. Quantification of individual risk.**

# The overall prediction-guided prevention strategy is shown in Fig. 4, and equations for individual risk score calculation in offspring of mothers with and without pre-conception obesity are provided in Table S5. Risk scores for higher-than-normal body mass index (BMI) growth in the early phase or late phase, respectively, can be calculated for each child at age 3 months, 1 year, and 2 years by adding the intercept and coefficients of the model. These parameters represent the adjusted contribution of each risk factor to higher-than-normal BMI growth. The final prediction models retained only those factors which contributed considerably to the risk score quantification (being still relevant after a cross validation-guided shrinkage of the coefficient).

The risk score calculated for an individual child is also called linear predictor in the terminology of logistic regression. It can be used to determine the risk in terms of probability as follows (“exp” being the exponential function):

$$Probability of higher-than-normal BMI growth=\frac{\exp\left( risk score \right)}{1+\exp\left( risk score \right)}$$

To calculate individual risk scores, each prenatal and postnatal variable in the risk quantification equations (Table S5) should be replaced by pre-defined values (0 or 1) depending on whether the condition stated is fulfilled (1) or not (0).

- 1. **Risk quantification in offspring of mother with obesity at well-child visits**

To illustrate how risk for higher-than-normal BMI growth in the early phase (between 6 months and 2 years) can be quantified, consider the following clinical case scenario of an offspring who was exposed to obesity in pregnancy:

**Initial risk quantification at age 3 months for developing higher-than-normal BMI growth in the early phase:**

A primiparous mother with class 3 obesity at conception, who had excessive gestational weight gain (GWG), developed gestational diabetes (GDM), did not smoke during pregnancy, and belonged to low/medium socioeconomic status (SES), gave birth to a boy with a large-for-gestational-age (LGA) birth weight and a BMI z-score of 1.01 SD at age 3 months. The boy was not fully breastfed (BF) at ages 1 month and 3 months. Note that “SGA” indicates “small-for-gestational-age” birth weight.

“Higher-than-normal BMI growth pattern” during the early phase (6 months to 2 years) = -2.094 + 0.036 * LGA + 0.892 * LGA * inadequate GWG - 0.671 * SGA * male sex + 0.185 * GDM positive * SGA + 0.129 * GDM positive * excessive GWG + 0.013 * GDM positive * maternal class 3 obesity - 0.429 * GDM positive * smoking during pregnancy + 0.204 * GDM positive * full BF at 3m + 0.147 * excessive GWG * male sex + 2.003 * BMI z-score > 1 SD at 3m + 0.246 * BMI z-score > 1 SD at 3m * SGA + 0.837 * BMI z-score > 1 SD at 3m * inadequate GWG + 0.388 * BMI z-score > 1 SD at 3m * primiparity + 0.142 * maternal class 2 obesity * LGA + 0.036 * maternal class 2 obesity * SGA + 0.093 * maternal class 2 obesity * primiparity + 0.195 * maternal class 2 obesity * full BF at 3m + 0.287 * maternal class 2 obesity * LGA + 0.183 * maternal class 3 obesity * excessive GWG - 0.193 * smoking during pregnancy * LGA + 0.222 * smoking during pregnancy * SGA + 0.211 * full BF at 3m * excessive GWG =

-2.094 + 0.036 * 1 + 0.892 * 1 * 0 - 0.671 * 0 * 1 + 0.185 * 1 * 0 + 0.129 * 1 * 1 + 0.013 * 1 * 1 - 0.429 * 1 * 0 + 0.204 * 1 * 0 + 0.147 * 1 * 1 + 2.003 * 1 + 0.246 * 1 * 0 + 0.837 * 1 * 0 + 0.388 * 1 * 1 + 0.142* 0 * 1 + 0.036 * 0 * 0 + 0.093 * 0 * 1 + 0.195 * 0 * 0 + 0.287 * 0 * 1 + 0.183 * 1 * 1 - 0.193 * 0 * 1 + 0.222 * 0 * 0 + 0.211 * 0 * 1 =

-2.094 + 0.036 + 0.129 + 0.013 + 0.147 + 2.003 + 0.388 + 0.183 = 0.805.

Calculating the odds of higher-than-normal BMI growth: exp(risk score) = exp(0.805) = 2.24.

Calculating the probability of higher-than-normal BMI growth: exp(risk score)/(1+exp(risk score) = 2.24/3.24 = 0.69.

We will now compare the calculated individual risk score (0.805) to the respective cut-off score value presented in Table 2 in the main text (-1.689). Since 0.805 is greater than -1.689, the child will be classified as belonging to the “higher-than-normal BMI growth pattern” risk group. The calculated risk probability of the child to develop “higher-than-normal BMI growth pattern” is 69%. The pediatrician should discuss preventive measures with the mother to reduce the risk score measured during the next assessment.

**First risk re-assessment at age 1 year for developing higher-than-normal BMI growth in the late phase:**

At the well-child visit at age 1 year, the risk of this offspring can be re-assessed using the respective risk quantification equation. The pediatrician obtains new information (after month 3) and learns that this child was not fully breastfed at age 6 months and has a BMI z-score of 1.50 SD at age 1 year.

“Higher-than-normal BMI growth pattern” during the late phase (3 years to 5 years) = -1.446 + 0.28 * LGA * inadequate GWG - 0.052 * SGA * male sex + 0.053 * GDM positive * low/medium SES + 0.648 * BMI z-score > 1 SD at 1y + 0.46 * BMI z-score > 1 SD at 1y * GDM positive + 0.59 * BMI z-score > 1 SD at 1y * excessive GWG + 0.122 * BMI z-score > 1 SD at 1y * primiparity + 0.352 * BMI z-score > 1 SD at 1y * male sex + 0.057 * BMI z-score > 1 SD at 1y * full BF at 1m + 0.311 * maternal class 3 obesity * full BF at 1m + 0.185 * smoking during pregnancy + 0.084 * smoking during pregnancy * LGA + 0.121 * smoking during pregnancy * low/medium SES + 0.551* full BF at 6m * LGA + 0.026 * full BF at 6m * smoking during pregnancy =

-1.446 + 0.28 * 1 * 0 - 0.052 * 0 * 1 + 0.053 * 1 * 1 + 0.648 * 1 + 0.46 * 1 * 1 + 0.59 * 1 * 1 + 0.122 * 1 * 1 + 0.352 * 1 * 1 + 0.057* 1 * 0 + 0.311 * 1 * 0 + 0.185 * 0 + 0.084 * 0 * 1 + 0.121 * 0 * 1 + 0.551 * 0 * 1 + 0.026 * 0 * 0 =

-1.446 + 0.053 + 0.648 + 0.46 + 0.59 + 0.122 + 0.352 = 0.779

Calculating the odds of higher-than-normal BMI growth: exp(risk score) = exp(0.779) = 2.18.

Calculating the probability of higher-than-normal BMI growth: exp(risk score)/(1+exp(risk score) = 2.18/3.18 = 0.69

We will now compare the calculated individual risk score (0.779) to the respective cut-off score value presented in Table 2 (-1.135). Since 0.779 is greater than -1.135, the child will still be classified as belonging to the “higher-than-normal BMI growth pattern” risk group during the late phase. The individual risk probability of this child to develop a “higher-than-normal BMI growth pattern” remains to be 69%. The pediatrician should continue to discuss preventive measures with the mother to reduce the risk score measured during the next assessment.

**Second risk re-assessment at age 2 years for developing higher-than-normal BMI growth in the late phase:**

At age 2 years, the risk of this offspring can be further re-assessed using the respective risk quantification equation. This child has a BMI z-score of 1.70 SD at age 2 years.

“Higher-than-normal BMI growth pattern” during the late phase (3 years to 5 years) = -1.995 + 0.112 * GDM positive * excessive GWG + 0.022 * GDM positive * low/medium SES + 0.109* GDM positive * smoking during pregnancy + 0.157 * GDM positive * full BF at 3m + 1.734 * BMI z-score > 1 SD at 2y - 0.097 * BMI z-score > 1 SD at 2y * SGA + 0.419 * BMI z-score > 1 SD at 2y * primiparity + 0.214 * BMI z-score > 1 SD at 2y * maternal class 3 obesity + 0.037 * BMI z-score > 1 SD at 2y * male sex + 0.142 * maternal class 3 obesity * full BF at 3m + 0.036 * smoking during pregnancy + 0.144 * full BF at 3m * LGA + 0.044 * full BF at 3m * excessive GWG + 0.015 * full BF at 3m * male sex + 0.531 * full BF at 6m * LGA + 0.14 * full BF at 6m * male sex =

-1.995 + 0.112 * 1 * 1 + 0.022 * 1 * 1 + 0.109 * 1 * 0 + 0.157 * 1 * 0 + 1.734 * 1 - 0.097 * 1 * 0 + 0.419 * 1 * 1 + 0.214 * 1 * 1 + 0.037 * 1 * 1 + 0.142 * 1 * 0 + 0.036 * 0 + 0.144 * 0 * 1 + 0.044 * 0 * 1 + 0.015 * 0 * 1 + 0.531 * 0 * 1 + 0.14 * 0 * 1 =

-1.995 + 0.112 + 0.022 + 1.734 + 0.419 + 0.214 + 0.037 = 0.543

Calculating the odds of higher-than-normal BMI growth: exp(risk score) = exp(0.543) = 1.72.

Calculating the probability of higher-than-normal BMI growth: exp(risk score)/(1+exp(risk score) = 1.72/2.72 = 0.63

We will now compare the calculated individual risk score (0.543) to the respective cut-off score value presented in Table 2 (-1.133). Since 0.543 is greater than -1.133, the child will continue to have a high risk of higher-than-normal BMI growth during the late phase and an individual risk probability of 63%. The pediatrician should continue to discuss preventive measures with the mother to reduce the child’s risk of overweight.

- 1. **Risk quantification in offspring of mother without obesity at well-child visits**

To illustrate how risk for higher-than-normal BMI growth in the early phase (between 6 months and 2 years) can be quantified, consider the following clinical case scenario of an offspring whose mother was normal weight in pregnancy:

**Initial risk quantification at age 3 months for developing higher-than-normal BMI growth in the early phase:**

A primiparous mother with normal weight, who gained adequate GWG, developed GDM, did not smoke during pregnancy, and belonged to a high SES, gave birth to a girl with an average-for-gestational-age (AGA) birth weight and a BMI z-score of 0.82 SD at age 3 months. The girl was fully breastfed at ages 1 month and 3 months.

“Higher-than-normal BMI growth pattern” during the early phase (6 months to 2 years) = -2.065 + 0.113 * LGA * primiparity + 1.717 * BMI z-score > 1 SD at 3m + 0.072 * BMI z-score > 1 SD at 3m * GDM positive + 0.08 * BMI z-score > 1 SD at 3m * inadequate GWG + 0.105 * BMI z-score > 1 SD at 3m * smoking during pregnancy - 0.064 * GDM positive - 0.076 * inadequate GWG * primiparity + 0.139 * maternal overweight + 1.731 * maternal overweight * LGA + 0.057 * maternal overweight * excessive GWG + 0.061 * full BF at 1m * smoking during pregnancy =

-2.065 + 0.113 * 0 * 0 + 1.717 * 0 + 0.072 * 0 * 1 + 0.08 * 0 * 0 + 0.105 * 0 * 0 - 0.064 * 1 - 0.076 * 0 * 0 + 0.139 * 0 + 1.731 * 0 * 0 + 0.057 * 0 * 0 + 0.061 * 0 * 0 =

-2.065 - 0.064 = -2.129

Calculating the odds of higher-than-normal BMI growth: exp(risk score) = exp(-2.129) = 0.12.

Calculating the probability of higher-than-normal BMI growth: exp(risk score)/(1+exp(risk score) = 0.12/1.12 = 0.11

We will now compare the calculated individual risk score (-2.129) to the respective cut-off score value presented in Table S4 (Additional file 1) (-2.065). Since -2.129 is lower than -2.065, the child has a low risk of developing higher-than-normal BMI growth (individual risk probability 11%) and will not be classified as belonging to the “higher-than-normal BMI growth pattern” risk group.

**First risk re-assessment at age 1 year for developing higher-than-normal BMI growth in the late phase:**

At age 1 year, the risk of this offspring can be re-assessed using the respective risk quantification equation, given that this child was fully breastfed at age 6 months and has a BMI z-score of 0.89 SD at age 1 year.

“Higher-than-normal BMI growth pattern” during late phase (3 years to 5 years) =

-1.874 + 0.22 * BMI z-score > 1 SD at 1y =

-1.874 + 0.22 * 0 = -1.874

Calculating the odds of higher-than-normal BMI growth: exp(risk score) = exp(-1.874) = 0.15.

Calculating the probability of higher-than-normal BMI growth: exp(risk score)/(1+exp(risk score) = 0.15/1.15 = 0.13

We will now compare the calculated individual risk score (-1.874) to the respective cut-off score value presented in Table S4 (Additional file 1) (-1.651). Since -1.874 is lower than -1.651 and the risk probability is 13%, the child will again not be classified as belonging to the “higher-than-normal BMI growth pattern” risk group.

**Second risk re-assessment at age 2 years for developing higher-than-normal BMI growth in the late phase:**

At age 2 years, the risk of this offspring can be further re-assessed using the respective risk quantification equation. This child has a BMI z-score of 0.92 SD at age 2 years.

“Higher-than-normal BMI growth pattern” during the late phase (3 years to 5 years) =

-1.895 + 0.23 * BMI z-score > 1 SD at 2y =

-1.895 + 0.23 * 0 = -1.895

Calculating the odds of higher-than-normal BMI growth: exp(risk score) = exp(-1.895) = 0.15.

Calculating the probability of higher-than-normal BMI growth: exp(risk score)/(1+exp(risk score) = 0.15/1.15 = 0.13

We will now compare the calculated individual risk score (-1.895) to the respective cut-off score value presented in Table S4 (Additional file 1) (-1.665). Since -1.895 is lower than -1.665 and the risk probability remains to be 13%, the child will again not be classified as belonging to the “higher-than-normal BMI growth pattern” risk group.

# **Supplementary figures**

**Figure S1. Influence of maternal obesity on offspring BMI growth outcomes.**


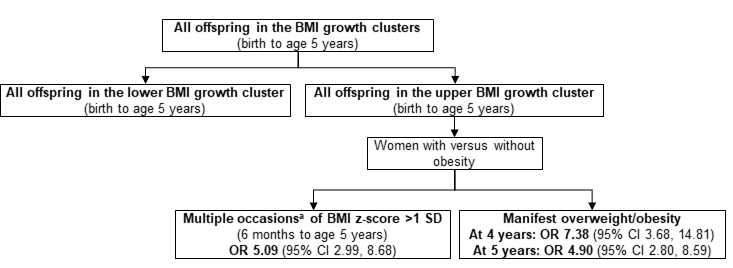


Shown are ORs and 95% CIs of the influence of maternal pre-conception obesity on BMI growth outcomes up to age 5 years in all offspring belonging to upper BMI growth clusters from the PEACHES cohort study. Values were derived from univariate logistic regression.

^a^The term “multiple occasions” was defined as having BMI z-scores >1 SD [51] at least 5 out of 6 times at the well-child visits at age 6 months, 1 year, 2 years, 3 years, 4 years, and 5 years.

BMI, body mass index; CI, confidence interval; OR, odds ratio; PEACHES, Programming of Enhanced Adiposity Risk in Childhood–Early Screening.

**Figure S2. Proportion of offspring in upper and lower BMI growth clusters according to birth weight category.**

**
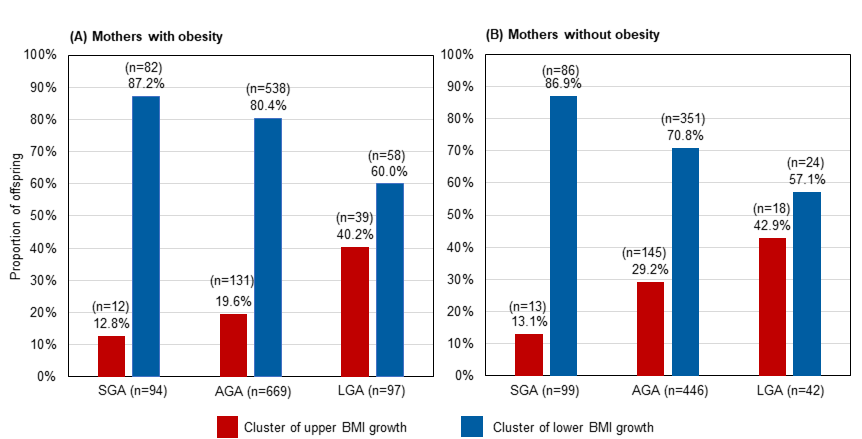
**

Shown are percentages in offspring of mothers with obesity (panel A) and without (panel B) enrolled in the PEACHES cohort study, according to their birth weight category for gestational age and sex.

AGA, average-for-gestational-age; BMI, body mass index; LGA, large-for-gestational-age; PEACHES, Programming of Enhanced Adiposity Risk in Childhood–Early Screening; SGA, small-for-gestational-age.

# **Figure S3. Mean BMI growth clusters by birth weight category in offspring of mothers with and without obesity.**

**
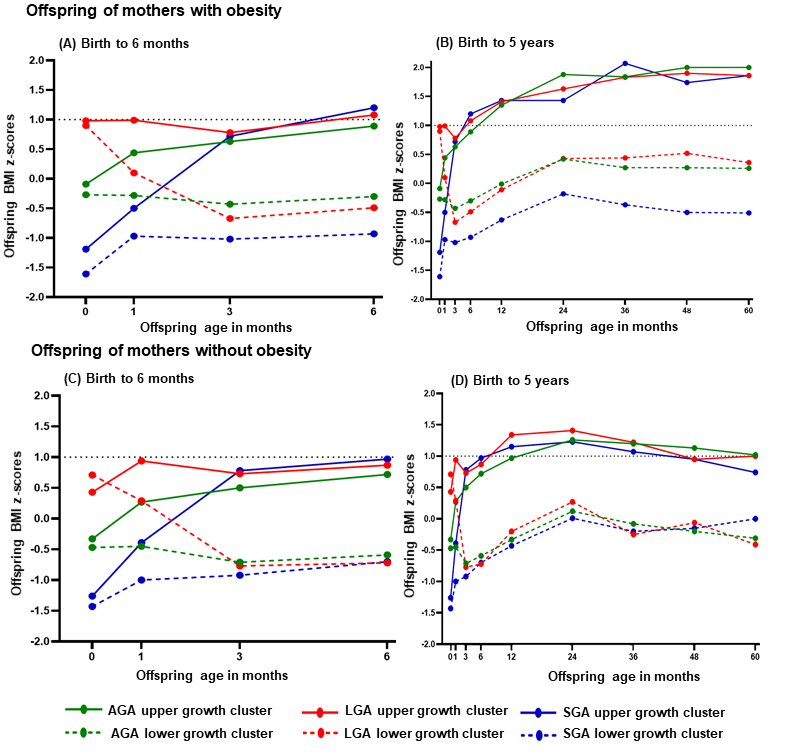
**

Shown are mean BMI z-score growth clusters from birth to age 6 months (panel A, C) and birth to age 5 years (panel B, D) by birth weight category for gestational age and sex in offspring of mothers with and without obesity enrolled in the PEACHES cohort study.

AGA, average-for-gestational-age; BMI, body mass index; LGA, large-for-gestational-age; PEACHES, Programming of Enhanced Adiposity Risk in Childhood–Early Screening; SGA, small-for-gestational-age.

# **Figure S4. Effects of prenatal and postnatal factors on BMI growth outcomes in offspring of mothers without obesity.**


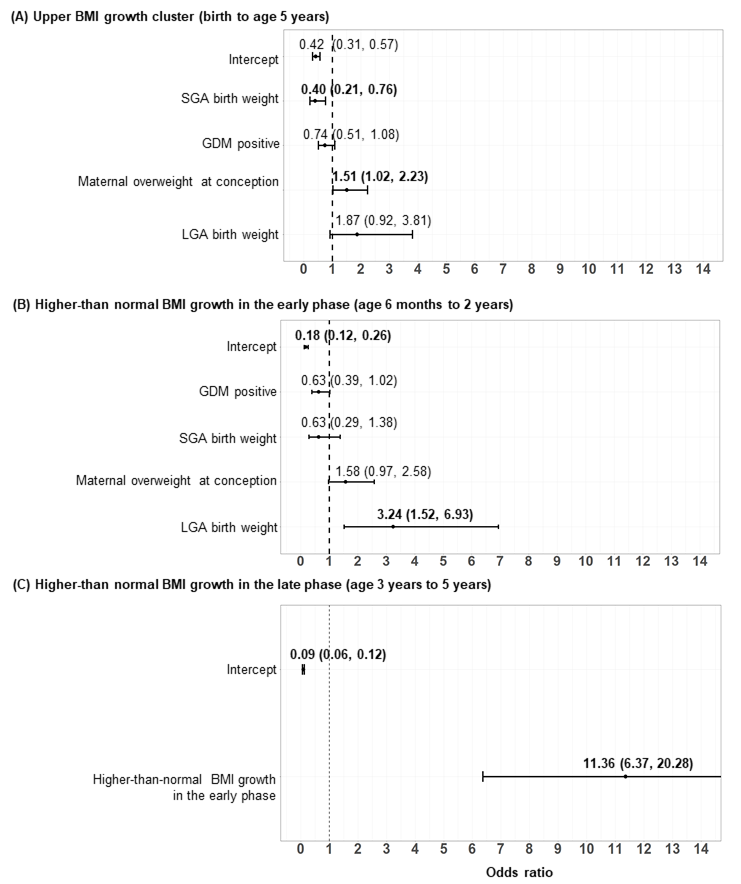


Shown are ORs and 95% CI of the influence of prenatal and postnatal factors on the development of an upper cluster of BMI growth (birth to age 5 years, panel A) and a “higher-than-normal BMI growth pattern”, defined as BMI z-score >1 SD [51] at least twice, during early phase (6 months to 2 years, panel B) and late phase (3 years to 5 years, panel C) in offspring of mothers without obesity enrolled in the PEACHES cohort study. Values were derived from multivariable logistic regression with stepwise backward selection. Only final models based on the lowest Akaike information criterion are presented. Included variables in all initial models were maternal pre-conception BMI group, total GWG, GDM, parity, smoking during pregnancy, sex, birth weight category for gestational age and sex, SES, breastfeeding status at 1 month. Additionally, for associations shown in panel C, “higher-than-normal BMI growth pattern” in the early phase was also included as an explanatory variable in the initial model.

BMI, body mass index; CI, confidence interval; GDM, gestational diabetes; GWG, gestational weight gain; LGA, large-for-gestational-age; OR, odds ratio; PEACHES, Programming of Enhanced Adiposity Risk in Childhood–Early Screening; SES, socioeconomic status; SGA, small-for-gestational-age.

**Figure S5. Calibration plots of prediction models for identifying a “higher-than-normal BMI growth pattern” in the validation cohort.**

**
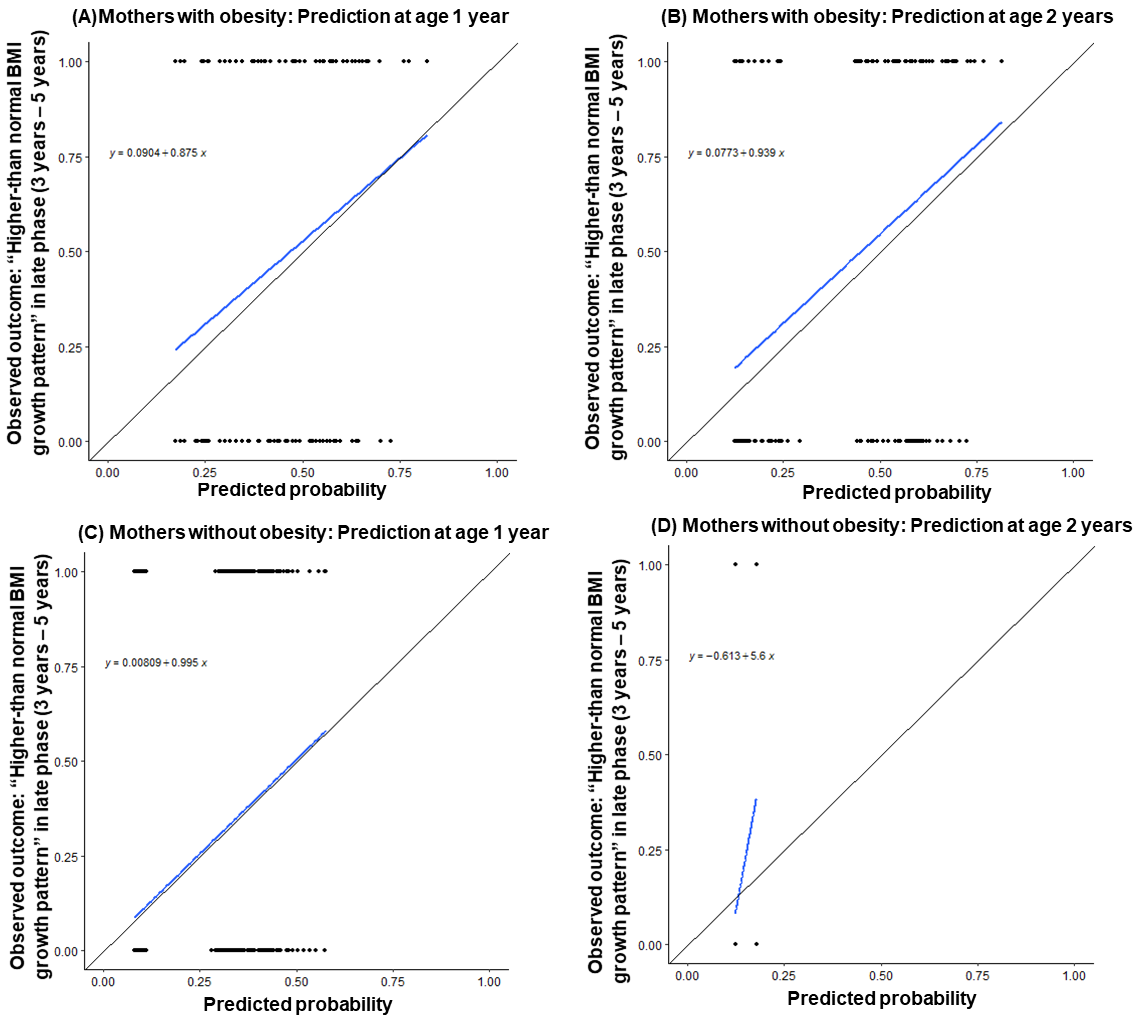
**

Shown are calibration curves (blue lines) and calibration slopes and intercepts for offspring of mothers with obesity (panel A, B) and without obesity (panel C, D) by the prediction models at age 1 year and 2 years. The diagonal grey lines represent the optimal prediction; the closer the model curve is to the diagonal line, the more accurate is the prediction. At the top of each graph, dots indicate presence of the outcome “higher-than-normal BMI growth pattern”, defined as BMI z-score >1 SD [51] at least twice, in the late phase (3 years to 5 years). At the bottom of each graph, dots indicate absence of the outcome “higher-than-normal BMI growth pattern” in the late phase. Calibration of models at age 3 months for “higher-than-normal BMI growth pattern” in the early phase (6 months to 2 years) could not be performed due to the lack of follow-up data at age 3 months in the validation cohort PEPO. BMI, body mass index; PEPO, PErinatal Prevention of Obesity.

**Supplementary tables**

| **Table S1. Offspring follow-up rates in the study populations.** | | | |
| --- | --- | --- | --- |
| **Offspring age** | **Follow-up anthropometric data** | **Discovery cohort: PEACHES** | **Validation cohort: PEPO** |
|  |  | **All (n=1,557)** | **All (n=9,874)** |
| **At birth** | Missing^a^ | 0 (0.0) | 38 (0.4) |
|  | Available | 1,557 (100.0) | 9,836 (99.6) |
|  | Total | 1,557 (100.0) | 9,874 (100.0) |
| **1 month** | Missing^a^ | 31 (2.0) | NA |
|  | Available | 1,526 (97.3) | NA |
|  | Total | 1,557 (100.0) | NA |
| **3 months** | Missing^a^ | 42 (2.7) | NA |
|  | Available | 1,515 (97.3) | NA |
|  | Total | 1,557 (100.0) | NA |
| **6 months** | Missing^a^ | 68 (4.4) | NA |
|  | Available | 1,489 (95.6) | NA |
|  | Total | 1,557 (100.0) | NA |
| **1 year** | Missing^a^ | 60 (3.9) | 502 (5.1) |
|  | Available | 1,497 (96.1) | 9,372 (94.9) |
|  | Total | 1,557 (100.0) | 9,874 (100.0) |
| **2 years** | Missing^a^ | 104 (6.7) | 627 (6.4) |
|  | Available | 1,453 (93.3) | 9,247 (93.6) |
|  | Total | 1,557 (100.0) | 9,874 (100.0) |
| **3 years** | Missing^a^ | 184 (11.8) | NA |
|  | Available | 1,373 (88.2) | NA |
|  | Total | 1,557 (100.0) | NA |
| **4 years** | Missing^a^ | 263 (17.0) | NA |
|  | Available | 1,281 (83.0) | NA |
|  | Total | 1,544 (100.0)^b^ | NA |
| **5 years** | Missing^a^ | 252 (20.0) | 307 (3.1) |
|  | Available | 1,008 (80.0) | 9,567 (96.9) |
|  | Total | 1,260 (100.0)^b^ | 9,874 (100.0) |
| **Total over all ages** | Missing^a^ | 1,004 (7.3) | 1,474 (3.7) |
|  | Available | 12,699 (92.7) | 38,022 (96.3) |
|  | Total | 13,703 (100.0) | 39,496 (100.0) |
| Values are n (%).  ^a^Missing data in the PEACHES cohort were due to loss to follow-up. Missing data in the PEPO cohort were due to lack of availability of data in the records of the regular well-child visits at the time of school entry health examination.  ^b^A total of 13 and 297 children enrolled in the PEACHES cohort were too young for the follow-up visit at age 4 and 5 years, respectively, and therefore were not included in the “total” category. Missing data were considered missing completely at random.  NA, not available; PEACHES, Programming of Enhanced Adiposity Risk in Childhood–Early Screening; PEPO, PErinatal Prevention of Obesity. | | | |

| **Table S2. Mean BMI z-scores by BMI growth cluster in offspring of mothers with and without obesity.** | | | | | | | | |
| --- | --- | --- | --- | --- | --- | --- | --- | --- |
| **Offspring follow-up** | **Upper BMI growth cluster** | | | | **Lower BMI growth cluster** | | | |
|  | **Mothers with obesity**^a^ | | **Mothers without obesity**^b^ | | **Mothers with obesity**^a^ | | **Mothers without obesity**^b^ | |
|  | N | Mean (95% CI) | N | Mean (95% CI) | N | Mean (95% CI) | N | Mean (95% CI) |
| At birth | 185 | 0.07 (-0.06, 0.21) | 183 | -0.33 (-0.47, -0.19) | 690 | -0.31 (-0.39, -0.24) | 472 | -0.62 (-0.71, -0.53) |
| 1 month | 185 | 0.50 (0.37, 0.62) | 182 | 0.28 (0.15, 0.42) | 687 | -0.33 (-0.40, -0.26) | 472 | -0.51 (-0.58, -0.43) |
| 3 months | 184 | 0.67 (0.54, 0.80) | 181 | 0.55 (0.41, 0.68) | 682 | -0.51 (-0.58, -0.44) | 468 | -0.77 (-0.85, -0.70) |
| 6 months | 181 | 0.95 (0.82, 1.08) | 176 | 0.76 (0.64, 0.88) | 672 | -0.37 (-0.44, -0.29) | 460 | -0.66 (-0.74, -0.58) |
| 1 year | 182 | 1.37 (1.25, 1.48) | 176 | 1.02 (0.91, 1.14) | 677 | -0.07 (-0.13, 0.00) | 462 | -0.38 (-0.46, -0.30) |
| 2 years | 174 | 1.79 (1.66, 1.93) | 171 | 1.27 (1.17, 1.37) | 665 | 0.38 (0.31, 0.44) | 443 | 0.07 (-0.01, 0.15) |
| 3 years | 168 | 1.85 (1.72, 1.99) | 165 | 1.19 (1.05, 1.33) | 621 | 0.23 (0.16, 0.30) | 419 | -0.14 (-0.21, -0.07) |
| 4 years^c^ | 155 | 1.97 (1.83, 2.11) | 146 | 1.10 (1.00, 1.20) | 588 | 0.24 (0.18, 0.31) | 392 | -0.25 (-0.32, -0.18) |
| 5 years^c^ | 124 | 1.96 (1.77, 2.16) | 111 | 0.99 (0.86, 1.13) | 461 | 0.24 (0.16, 0.32) | 312 | -0.35 (-0.43, -0.28) |
| Values are mean and 95% CI in offspring of mothers with and without obesity enrolled in the PEACHES cohort study.  ^a^Of a total of 887 children included for cluster analysis, 875 children could be categorized into longitudinal BMI growth clusters based on an adequate number of data points.  ^b^Of a total of 670 children included for cluster analysis, 655 children could be categorized into clusters based on an adequate number of data points.  ^c^A total of 276 and 549 children enrolled in the PEACHES cohort were not included in the cluster analysis at age 4 and 5 years, respectively, because of follow-up not yet due (age 4 years: n=13, age 5 years: n=297) or missing data due to loss to follow-up (age 4 years: n=263, age 5 years: n=252). Missing data were considered missing completely at random.  BMI, body mass index; CI, confidence interval; PEACHES, Programming of Enhanced Adiposity Risk in Childhood–Early Screening. | | | | | | | | |

| **Table S3. Offspring BMI growth dynamics in consecutive life phases after birth following exposure to gestational obesity.** | | | | | | | |
| --- | --- | --- | --- | --- | --- | --- | --- |
|  |  | **Late phase: 3 years – 5 years** | | | | | |
|  |  | **Mothers with obesity (n=584)** | | | **Mothers without obesity (n=428)** | | |
|  | BMI z-score category | ≤1 SD^a^ | >1 SD^b^ | “Higher-than-normal BMI growth pattern”^c^ | ≤1 SD^a^ | >1 SD^b^ | “Higher-than-normal BMI growth pattern”^c^ |
| **Early phase:**  **6 months – 2 years** | ≤1 SD^a^ | 263 (45.0) | 39 (6.7) | 25 (4.3) | 248 (57.9) | 17 (4.0) | 7 (1.6) |
|  | >1 SD^b^ | 22 (3.8) | 44 (7.5) | 70 (12.0) | 20 (4.7) | 39 (9.1) | 28 (6.5) |
|  | “Higher-than-normal BMI growth pattern”^c^ | 9 (1.5) | 16 (2.7) | 96 (16.4) | 5 (1.2) | 27 (6.3) | 37 (8.7) |
|  | All patterns | 294 (50.3) | 99 (16.9) | 191 (32.7) | 273 (63.8) | 83 (19.4) | 72 (16.8) |
| Values are n (%) in offspring enrolled in the PEACHES cohort study. Only children with complete data on BMI z-scores in both the early and late phase are presented.  ^a^Includes values for categories “normal range” (≥-2 to ≤1 SD) and a minor proportion of children with <-2 SD [72].  ^b^BMI z-score >1 SD defined as occurring once. Includes values for categories “at risk of overweight” (>1 to ≤2 SD), overweight (>2 to ≤3 SD), and obesity (>3 SD) [51].  ^c^“Higher-than-normal BMI growth pattern” defined as BMI z-score >1 SD [51] at least twice.  BMI, body mass index; PEACHES, Programming of Enhanced Adiposity Risk in Childhood–Early Screening. | | | | | | | |

| **Table S4. Predictive performance of a sequential algorithm to identify higher-than-normal BMI growth in offspring of mothers without obesity.** | | | | | |
| --- | --- | --- | --- | --- | --- |
| **Predictive parameter** | **Prediction at age 3 months**^a^ | **Prediction at age 1 year**^b^ | | **Prediction at age 2 years**^c^ | |
|  | **Higher-than-normal BMI growth in early phase (6 months – 2 years)** | **Higher-than-normal BMI growth in late phase (3 years – 5 years)** | | **Higher-than-normal BMI growth in late phase (3 years – 5 years)** | |
|  | **Discovery cohort** | **Discovery cohort** | **Validation cohort** | **Discovery cohort** | **Validation cohort** |
| N | 567 | 515 | 6521 | 513 | 6432 |
| AUROC | 0.68 (0.66, 0.71) | 0.69 (0.67, 0.72) | 0.65 | 0.77 (0.75, 0.79) | 0.71 |
| Cut-off score value^d^ | -2.065 | -1.651 | NA | -1.665 | NA |
| Prevalence, n (%) | 82 (14.5) | 71 (13.8) | 949 (14.6) | 71 (13.8) | 924 (14.4) |
| Sensitivity, % | 75.6 (65.7, 83.4) | 49.3 (38.3, 60.4) | 42.0 (39.3, 44.8) | 73.2 (62.2, 81.9) | 65.1 (62.4, 67.7) |
| Specificity, % | 59.0 (48.4, 68.9) | 88.1 (84.2, 91.1) | 75.1 (72.8, 77.3) | 80.1 (72.1, 86.3) | 72.8 (70.0, 75.4) |
| Positive predictive value, % | 23.3 (17.4, 30.7) | 40.2 (28.2, 52.5) | 22.6 (20.0, 25.5) | 37.5 (26.7, 49.4) | 29.0 (26.2, 32.0) |
| Negative predictive value, % | 93.6 (89.5, 96.2) | 91.5 (89.4, 93.4) | 88.2 (87.4, 89.0) | 94.8 (92.1, 96.7) | 92.4 (91.6, 93.2) |
| Positive likelihood ratio | 1.84 (1.27, 2.68) | 4.14 (2.42, 6.82) | 1.69 (1.44, 1.97) | 3.68 (2.23, 5.97) | 2.39 (2.08, 2.75) |
| Negative likelihood ratio | 0.41 (0.24, 0.71) | 0.58 (0.43, 0.73) | 0.77 (0.71, 0.83) | 0.33 (0.21, 0.52) | 0.48 (0.43, 0.54) |
| We used the PEACHES cohort study as the discovery cohort and the PEPO cohort study as the external validation cohort for calculation of the individual child’s risk of a “higher-than-normal BMI growth pattern” (BMI z-score >1 SD [51] at least twice). Values are predictive parameters and their 95% CI.  ^a^Potential predictors included: maternal pre-conception BMI group, total GWG, GDM, parity, smoking during pregnancy, sex, birth weight category for gestational age and sex, SES, breastfeeding status at 1 month, breastfeeding status at 3 months, and BMI z-score >1 SD at age 3 months. External validation of models at age 3 months could not be performed due to the lack of follow-up data at age 3 months in the validation cohort PEPO.  ^b^Potential predictors included: maternal pre-conception BMI group, total GWG, GDM, parity, smoking during pregnancy, sex, birth weight category for gestational age and sex, SES, breastfeeding status at 1 month, breastfeeding status at 3 months, breastfeeding status at 6 months, and BMI z-score >1 SD at age 1 year. External validation of models at age 1 year was performed in the validation cohort PEPO.  ^c^Potential predictors included: maternal pre-conception BMI group, total GWG, GDM, parity, smoking during pregnancy, sex, birth weight category for gestational age and sex, SES, breastfeeding status at 1 month, breastfeeding status at 3 months, breastfeeding status at 6 months, and BMI z-score >1 SD at age 2 years. External validation of models at age 2 years was performed in the validation cohort PEPO.  ^d^Offspring with a risk score above or equal to the respective cut-off score value are considered to be at risk of developing a “higher-than-normal BMI growth pattern”. The cut-off value of the score was optimized to avoid false-negative findings (sensitivity), which resulted in negative cut-off score values.  AUROC, area under the receiver operating characteristic; BMI, body mass index; CI, confidence interval; GDM, gestational diabetes; GWG, gestational weight gain; NA, not applicable; PEACHES, Programming of Enhanced Adiposity Risk in Childhood–Early Screening; PEPO, PErinatal Prevention of Obesity; SES, socioeconomic status. | | | | | |

| **Table S5. Scoring system for quantification of risk of higher-than-normal BMI growth in young offspring.** | | |
| --- | --- | --- |
| **Prediction time point and outcome**^a^ | **Offspring of mothers with obesity** | **Offspring of mothers without obesity** |
| Prediction at age 3 months: Higher-than-normal BMI growth in early phase (6 months – 2 years) | -2.094 + 0.036 * LGA + 0.892 * LGA * inadequate GWG - 0.671 * SGA * male sex + 0.185 * GDM positive * SGA + 0.129 * GDM positive * excessive GWG + 0.013 * GDM positive * maternal class 3 obesity - 0.429 * GDM positive * smoking during pregnancy + 0.204 * GDM positive * full BF at 3m + 0.147 * excessive GWG * male sex + 2.003 * BMI z-score > 1 SD at 3m + 0.246 * BMI z-score > 1 SD at 3m * SGA + 0.837 * BMI z-score > 1 SD at 3m * inadequate GWG + 0.388 * BMI z-score > 1 SD at 3m * primiparity + 0.142 * maternal class 2 obesity * LGA + 0.036 * maternal class 2 obesity * SGA + 0.093 * maternal class 2 obesity * primiparity + 0.195 * maternal class 2 obesity * full BF at 3m + 0.287 * maternal class 2 obesity * LGA + 0.183 * maternal class 3 obesity * excessive GWG - 0.193 * smoking during pregnancy * LGA + 0.222 * smoking during pregnancy * SGA + 0.211 * full BF at 3m * excessive GWG | -2.065 + 0.113 * LGA * primiparity + 1.717 * BMI z-score > 1 SD at 3m + 0.072 * BMI z-score > 1 SD at 3m * GDM positive + 0.08 * BMI z-score > 1 SD at 3m * inadequate GWG + 0.105 * BMI z-score > 1 SD at 3m * smoking during pregnancy - 0.064 * GDM positive - 0.076 * inadequate GWG * primiparity + 0.139 * maternal overweight + 1.731 * maternal overweight * LGA + 0.057 * maternal overweight * excessive GWG + 0.061 * full BF at 1m * smoking during pregnancy |
| Prediction at age 1 year: Higher-than-normal BMI growth in late phase (3 years – 5 years) | -1.446 + 0.28 * LGA * inadequate GWG - 0.052 * SGA * male sex + 0.053 * GDM positive * low/medium SES + 0.648 * BMI z-score > 1 SD at 1y + 0.46 * BMI z-score > 1 SD at 1y * GDM positive + 0.59 * BMI z-score > 1 SD at 1y * excessive GWG + 0.122 * BMI z-score > 1 SD at 1y * primiparity + 0.352 * BMI z-score > 1 SD at 1y * male sex + 0.057 * BMI z-score > 1 SD at 1y * full BF at 1m + 0.311 * maternal class 3 obesity * full BF at 1m + 0.185 * smoking during pregnancy + 0.084 * smoking during pregnancy * LGA + 0.121 * smoking during pregnancy * low/medium SES + 0.551* full BF at 6m * LGA + 0.026 * full BF at 6m * smoking during pregnancy | -1.874 + 0.22 * BMI z-score > 1 SD at 1y |
| Prediction at age 2 years: Higher-than-normal BMI growth in late phase (3 years – 5 years) | -1.995 + 0.112 * GDM positive * excessive GWG + 0.022 * GDM positive * low/medium SES + 0.109* GDM positive * smoking during pregnancy + 0.157 * GDM positive * full BF at 3m + 1.734 * BMI z-score > 1 SD at 2y - 0.097 * BMI z-score > 1 SD at 2y * SGA + 0.419 * BMI z-score > 1 SD at 2y * primiparity + 0.214 * BMI z-score > 1 SD at 2y * maternal class 3 obesity + 0.037 * BMI z-score > 1 SD at 2y * male sex + 0.142 * maternal class 3 obesity * full BF at 3m + 0.036 * smoking during pregnancy + 0.144 * full BF at 3m * LGA + 0.044 * full BF at 3m * excessive GWG + 0.015 * full BF at 3m * male sex + 0.531 * full BF at 6m * LGA + 0.14 * full BF at 6m * male sex | -1.895 + 0.23 * BMI z-score > 1 SD at 2y |
| ^a^The equations can be used for sequential individual risk quantification of a “higher-than-normal BMI growth pattern” (BMI z-score >1 SD [51] at least twice) in offspring of mothers with or without pre-conception obesity separately. The prenatal and postnatal variables in the risk quantification equations should be replaced by pre-defined values (0 or 1) depending on whether the condition stated is fulfilled (1) or not (0). The calculated risk score should be compared to the respective cut-off score value (Table 2). Offspring with a risk score above or equal to the respective cut-off are considered to be at risk of developing a “higher-than-normal BMI growth pattern”. Details on calculating individual risk probabilities and use of individual risk score calculations along with clinical case scenarios are provided in the Text S3 (Additional file 1).  BMI, body mass index; BF, breastfeeding; GDM, gestational diabetes; GWG, gestational weight gain; m, month(s); LGA, large-for-gestational-age; SES, socioeconomic status; SGA, small-for-gestational-age; y, year(s). | | |
